# Genome variation and LTR-RT analyses of an ancient peach landrace reveal mechanism of blood-flesh fruit color formation and fruit maturity date advancement

**DOI:** 10.1093/hr/uhad265

**Published:** 2023-12-19

**Authors:** Jiao Wang, Ke Cao, Yong Li, Jinlong Wu, Wenqing Li, Qi Wang, Gengrui Zhu, Weichao Fang, Changwen Chen, Xinwei Wang, Wenxuan Dong, Weisheng Liu, Lirong Wang

**Affiliations:** The Key Laboratory of Biology and Genetic Improvement of Horticultural Crops (Fruit TreeBreeding Technology), Ministry of Agriculture, Zhengzhou Fruit Research Institute, Chinese Academy of Agricultural Sciences, Zhengzhou 450009, China; The Key Laboratory of Biology and Genetic Improvement of Horticultural Crops (Fruit TreeBreeding Technology), Ministry of Agriculture, Zhengzhou Fruit Research Institute, Chinese Academy of Agricultural Sciences, Zhengzhou 450009, China; The Key Laboratory of Biology and Genetic Improvement of Horticultural Crops (Fruit TreeBreeding Technology), Ministry of Agriculture, Zhengzhou Fruit Research Institute, Chinese Academy of Agricultural Sciences, Zhengzhou 450009, China; The Key Laboratory of Biology and Genetic Improvement of Horticultural Crops (Fruit TreeBreeding Technology), Ministry of Agriculture, Zhengzhou Fruit Research Institute, Chinese Academy of Agricultural Sciences, Zhengzhou 450009, China; The Key Laboratory of Biology and Genetic Improvement of Horticultural Crops (Fruit TreeBreeding Technology), Ministry of Agriculture, Zhengzhou Fruit Research Institute, Chinese Academy of Agricultural Sciences, Zhengzhou 450009, China; The Key Laboratory of Biology and Genetic Improvement of Horticultural Crops (Fruit TreeBreeding Technology), Ministry of Agriculture, Zhengzhou Fruit Research Institute, Chinese Academy of Agricultural Sciences, Zhengzhou 450009, China; The Key Laboratory of Biology and Genetic Improvement of Horticultural Crops (Fruit TreeBreeding Technology), Ministry of Agriculture, Zhengzhou Fruit Research Institute, Chinese Academy of Agricultural Sciences, Zhengzhou 450009, China; The Key Laboratory of Biology and Genetic Improvement of Horticultural Crops (Fruit TreeBreeding Technology), Ministry of Agriculture, Zhengzhou Fruit Research Institute, Chinese Academy of Agricultural Sciences, Zhengzhou 450009, China; The Key Laboratory of Biology and Genetic Improvement of Horticultural Crops (Fruit TreeBreeding Technology), Ministry of Agriculture, Zhengzhou Fruit Research Institute, Chinese Academy of Agricultural Sciences, Zhengzhou 450009, China; The Key Laboratory of Biology and Genetic Improvement of Horticultural Crops (Fruit TreeBreeding Technology), Ministry of Agriculture, Zhengzhou Fruit Research Institute, Chinese Academy of Agricultural Sciences, Zhengzhou 450009, China; College of Horticulture, Shenyang Agricultural University, Shenyang 110866, China; Liaoning Institute of Pomology, Yingkou 115009, Liaoning, China; The Key Laboratory of Biology and Genetic Improvement of Horticultural Crops (Fruit TreeBreeding Technology), Ministry of Agriculture, Zhengzhou Fruit Research Institute, Chinese Academy of Agricultural Sciences, Zhengzhou 450009, China

## Abstract

Peach (*Prunus persica*) landrace has typical regional characteristics, strong environmental adaptability, and contains many valuable genes that provide the foundation for breeding excellent varieties. Therefore, it is necessary to assemble the genomes of specific landraces to facilitate the localization and utilization of these genes. Here, we *de novo* assembled a high-quality genome from an ancient blood-fleshed Chinese landrace Tianjin ShuiMi (TJSM) that originated from the China North Plain. The assembled genome size was 243.5 Mb with a contig N50 of 23.7 Mb and a scaffold N50 of 28.6 Mb. Compared with the reported peach genomes, our assembled TJSM genome had the largest number of specific structural variants (SVs) and long terminal repeat-retrotransposons (LTR-RTs). Among the LTR-RTs with the potential to regulate their host genes, we identified a 6688 bp LTR-RT (named it blood TE) in the promoter of NAC transcription factor-encoding *PpBL*, a gene regulating peach blood-flesh formation. The blood TE was not only co-separated with the blood-flesh phenotype but also associated with fruit maturity date advancement and different intensities of blood-flesh color formation. Our findings provide new insights into the mechanism underlying the development of the blood-flesh color and determination of fruit maturity date and highlight the potential of the TJSM genome to mine more variations related to agronomic traits in peach fruit.

## Introduction

Peach (*Prunus persica*) belongs to the Rosaceae family and has been cultivated for more than 4000 years in China [1]. It is the representative diploid species with 2n = 16 chromosomes and has a small genome size of about 200–300 Mb, which makes peach a desirable model species for genome study in the Rosaceae family [2]. The first peach genome Lovell was first reported in 2013 with an assembly size of 224.6 Mb [3]. Subsequently, this genome was improved by increasing the number of oriented sequences, correcting misassemblies, and improving base accuracy, in 2017 [4]. The genome Lovell has enabled rapid progress in peach gene detection, variation identification among different accessions, and evolutionary process analysis [5–9]. For instance, a total of 4 567 069 single nucleotide polymorphisms (SNPs) were identified among 84 peach accessions (10 wild relatives and 74 cultivated varieties). A subsequent phylogenetic tree constructed based on the SNPs/genotypes reveals that the edible and ornamental peach might originate from a single domesticated ancestor [5]. In addition, 202 273 structural variants (SVs) have been identified from 336 peach accessions after the alignment of Illumina sequencing reads to Lovell reference genome, and subsequently, some SVs have been found to be associated with 26 agronomic traits. Among these SVs, a 1.67 Mb insert containing a gene (*PpOFP1*) is highly associated with fruit shape [6].

Although the genome Lovell has promoted the aforementioned research, it is insufficient to represent the whole variation information of peach accessions, thus limiting new gene mining and variation discoveries. Recent studies have reported that an extensive range of genetic variations can be identified by comparing different assembled genomes with the reference genome. The related research has made great progress in several species, such as soybean, rice, apple, sorghum, and pigeon pea [10–14]. In peach, the new genomes of three cultivars including Chinese Cling, LHSM, and RYP1 have been reported since 2019 [15–17]. The first two cultivars belong to the Yangtze River middle and lower reaches group, and they are characterized by white flesh color, pleasant, sweet, and low-acid flavors in fruits, which are similar to those of the typical juicy honey peach (ShuiMi Tao in Chinese). The RYP1 cultivar is an improved cross variety between ‘Huan Xiang’ and ‘Rui Pan 5’, and it is characterized by white flesh color, sweet flavor and flat-fruit shape. In China, there are seven major groups for peach landraces [18], in which the accessions are characterized by specific botanical and biological traits. These traits provide valuable information for breeding peach varieties and for assembling a genome for a typical landrace.

**Table 1:** Statistics of assembled TJSM and four previously reported peach genomes.

Index	TJSM	Lovell v2.0	Chinese Cling	LHSM	RYP1
Total assembly size	243.5 Mb	227.4 Mb	247.3 Mb	257.2 Mb	239.1 Mb
Contig N50	23.7 Mb	225.4 kb	14.3 Mb	5.17 Mb	11.5 Mb
Number of contigs	130	2525	300	243	87
Scaffold N50	28.6 Mb	27.4 Mb	29.7 Mb		
Number of scaffolds	118	191	135		
Sequences anchored to chromosomes	237.6 Mb	225.7 Mb	240.3 Mb	246.0 Mb	235.0 Mb
Complete BUSCOs	98.9%	96.8%	96.4%	97.4%	97.4%
GC content	37.94%	37.05%	37.59%	37.57%	37.6%
Number of gene models	23 489	26 783	26 335	35 215	32 604
Mean transcript length	2697.3 bp	2215 bp	2632.3 bp	2175 bp	2223 bp
Repetitive sequences	118.45 Mb/48.64%	101.99 Mb/44.85%	114.66 Mb/46.36%	118.35 Mb/46.01%	115.01 Mb/48.11%

Here, we assembled a high-quality genome from an ancient blood-fleshed landrace Tianjin ShuiMi (TJSM) belonging to the China North Plain group. We performed a gene family clustering analysis of eight species genomes and conducted a variant identification based on the alignment of 4 genomes (TJSM, Chinese Cling, LHSM, and RYP1) with Lovell as the reference genome. We also investigated the characteristics of LTR-RTs in the TJSM genome and further explored the effect of a 6688 bp LTR-RT on blood-flesh formation and fruit maturity period advancement. Our work on genome assembly and analysis lays a foundation for exploring the genes controlling agronomic traits in the China North Plain group and deepens our understanding of the effects of transposons on phenotypic diversity of the peach fruit.

## Results

### Section 1: comparative analyses of assembled genome (TJSM) with other genomes

#### *De novo* genome sequencing and assembly

The Chinese landrace TJSM was sequenced using Illumina high-throughput, PacBio single molecule real time (SMRT), and Chromosome Conformation Capture-3C (Hi-C) sequencings, and 10.7 Gb Illumina reads, 200 Gb PacBio subreads, and 15.5 Gb Hi-C data, respectively, were obtained (Table S1, see online supplementary material). The 21 K-mer analysis of Illumina-sequencing data revealed that the TJSM genome was characterized by low levels of heterozygosity (0.26%). Based on the PacBio sequencing data, a genome with a size of approximately 243.54 Mb was *de novo* assembled, and this genome was comprised of 130 contigs and 118 scaffolds with a contig N50 of 23.66 Mb and a scaffold N50 of 28.64 Mb. This scaffold N50 value of TJSM was higher than that of Lovell v2.0 (27.4 Mb), LHSM (5.17 Mb), and RYP1 (11.5 Mb) and similar to that of Chinese Cling (29.7 Mb) (Table 1). To improve the accuracy of contig clustering and ordering in the genome, we employed Hi-C data for a subsequent scaffold assembly (Fig. S2, see online supplementary material). Finally, 97.57% of the total assembled genome was anchored on eight pseudo-chromosomes (Table S2, see online supplementary material), which was similar to that of Lovell v2.0 (99.2%), Chinese Cling (97.1%), LHSM (95.9%), and RYP1 (98.3%). Five strategies were selected to evaluate the genome assembly quality and completeness. Among 1614 of the total BUSCO group genes, 98.9% were identified in the TJSM genome (Table S3, see online supplementary material). In the GC-depth distribution, all scattered points were clustered together without any left–right partitioning (Fig. S3, see online supplementary material). Further, the Illumina reads were aligned to the genome assembled from PacBio reads, and the results showed that the alignment rate and the coverage rate reached up to 99.47% and 99.88%, respectively (Table S4, see online supplementary material). The assembly parameters of TJSM genome such as scaffold N50, complete BUSCOs, GC content, repetitive sequences, and contigN50 were similar to or higher than those of other genomes (Table 1). Good collinearity between the reference genome Lovell and our assembled genome TJSM indicated the reliability of Hi-C assembly quality (Fig. S4, see online supplementary material). The above results suggested that the TJSM genome had a high assembly quality and completeness.

#### Genome annotation

The TJSM genome annotation included four steps, namely, repeat sequence prediction, gene structure prediction, gene function annotation, and non-coding RNA prediction. The homologous sequence prediction and *ab initio* prediction results showed that approximately 118.45 Mb repeat sequences were identified from the TJSM genome, accounting for 48.64% in the whole genome (Table S5, see online supplementary material). Of these repeat sequences, 35.06 Mb and 23.04 Mb of LTR-RTs and DNA transposons were identified, accounting for approximately 14.40% and 9.56% in the whole genome, respectively. Combining with transcriptional data, homologous prediction, and *ab initio* prediction, we identified 23 488 protein-coding genes from the TJSM genome (Table S6, see online supplementary material). Finally, approximately 240 miRNAs, 543tRNAs, 4797 rRNAs, and 149 snRNAs were predicted in the TJSM genome using tRNA scan-SE software by comparing with a known non-coding RNA database (Rfam) (Table S7, see online supplementary material).

#### Genome comparison

**Figure 1:**
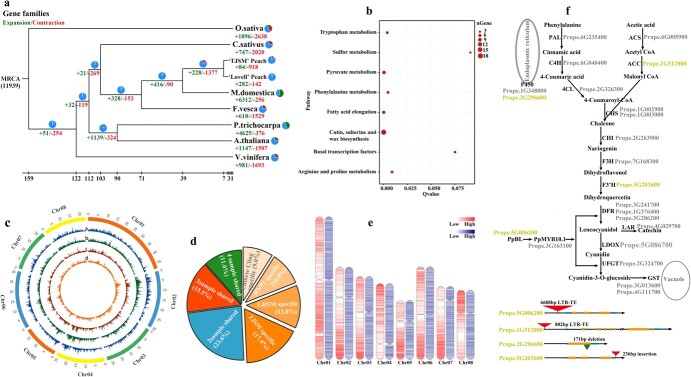
Gene families, SVs, and LTR-RT_S_ in TJSM genome. **a** Determination of expansion and contraction gene families of eight species. The green and red numbers represent the expansion and contraction gene families, respectively. MRCA indicates the most recent common ancestor. **b** Kyoto Encyclopedia of Genes and Genomes (KEGG) enrichment analysis of expanded gene families in TJSM genome. **c** Distribution of SVs identified in different peach genomes. The higher the broken line, the higher the SVs density was detected in the area. a, b, c, and d indicate four peach genomes TJSM, LHSM, RYP1, and Chinese Cling, respectively. **d** Pie chart of SVs identified by comparing Chinese Cling, LHSM, RYP1, and TJSM genomes with Lovell. **e** Distribution of genes and LTR-RTs in TJSM genome. The left red column indicates gene distribution on chromosome. The right blue column indicates LTR-RTs on chromosome. **f** Distribution of TJSM-specific SVs and the TJSM-specific LTR-RT_S_ on anthocyanin pathway genes.

To explore the relationship between gene families in the TJSM and specific agronomic traits, genome comparison analyses were performed among eight plant species, including *Malus domestica*, *P. persica*, *Fragaria ananassa*, *Arabidopsis thaliana*, *Vitis vinifera*, *Oryza sativa*, *Cucumis sativus*, and *Populus trichocarpa*. A total of 21 718 gene families were clustered, of which 8738 gene families and 1324 single-copyortholog gene families were shared by eight species (Fig. S5, see online supplementary material). Of the clustered 21 718 gene families, 161 were unique to the TJSM genome (Table S8, see online supplementary material). Expansion and contraction analyses of these gene families were conducted among these eight plant species. The results showed that the most recent common ancestor contained 11 939 gene families. The comparison of the gene families of the eight plant species found that a total of 918 gene families in the TJSM genome were contracted, of which 912 exhibited the significant contraction (*P* < 0.05) (Fig. 1a). A total of 84 gene families in the TJSM genome were expanded, of which 78 were significantly expanded (*P* < 0.05) (Fig. 1a). Of these 78 expanded gene families, five genes were enriched in phenylalanine metabolism synthesis pathway (Fig. 1b; Table S9, see online supplementary material). Because phenylalanine is a precursor to anthocyanin synthesis [19], these five expanded genes might be involved in the blood-flesh formation of TJSM species. Other expanded genes were mainly enriched in cutin, suberine, and wax biosynthesis, fatty acid elongation, and pyruvate metabolism pathways (Fig. 1b). The contracted genes were mainly enriched in ABC transporters, ether lipid metabolism, and sesquiterpenoid and triterpenoid biosynthesis pathways (Fig. S6, see online supplementary material).

#### Identification of genome variation in four peach varieties

To elucidate the similarities and differences in variation among different peach genomes, our assembled TJSM genome and three previously reported genomes (Chinese Cling, LHSM, and RYP1) were aligned with the reference genome Lovell. The results showed that a total of 2 338 330 SNPs and 1 112 185 small insertions/deletions (indels) were identified (Table S10, see online supplementary material), with many of them present in intergenic, promoter, and intronic regions. Of these SNPs and Indels, LHSM exhibited the largest number, followed by TJSM, Chinese Cling, and RYP1 (Figs S7 andS8 see online supplementary material)**.** A total of 38 327 structural variants (SVs) were identified with the sum SV length of approximately 207 900 613 bp for four genomes and a mean length of 51 975 153 bp for each genome (Table S10, see online supplementary material). These SVs had similar distribution among four genomes, but displayed visible difference in multiple regions of chromosomes (Fig. 1c). Subsequently, these identified SVs were merged into 14 060 non-redundant SVs, and of these non-redundant SVs, 11.6% was shared by four genomes; 15.5% was shared by three genomes; 23.6% was shared by two genomes; and 49.3% were specific to four genomes. Of these four genomes, TJSM exhibited the highest proportion (17.4%) of specific SVs, followed by LHSM (12.8%), RYP1 (10.0%), and Chinese Cling (9.0%) (Fig. 1d). The gene distribution characteristics of these specific SVs were similar among the four genomes, with many specific SVs present in intergenic, exonic, and promoter regions. However, the largest number of these specific SVs was observed in TJSM, followed by LHSM, RYP1, and Chinese Cling (Fig. S9, see online supplementary material). These results indicated that the TJSM genome had more specific SVs than other genomes. Because SVs had the potential to affect gene expression and functions [20], they might provide a source for the introduction of large numbers of new variations in peach breeding.

Gene ontology (GO) analysis showed that genes with specific ANPs in the TJSM, Chinese Cling, LHSM, and RYP1 genomes were mainly assigned to GO terms such as ‘hydrolase activity and phosphatidylinositol-3,5-bisphosphate phosphatase activity’, ‘lipase activity’, ‘DGTP metabolic process and purine nucleoside triphosphate catabolic process’ and ‘tryptophan catabolic process to kynurenine and indole-containing compound catabolic process’, respectively (Table S11, see online supplementary material). These indicated that SNPs specific to these four genomes may regulate genes with different functions. Genes with specific indels in the four genomes were not assigned to GO terms. In addition, genes with specific SV distribution in the TJSM genome were mainly assigned to GO terms such as cell death, host programmed cell death induced by symbiont, immune response, immune system process, innate immune response, plant-type hypersensitive response, and programmed cell death (Fig. S10, see online supplementary material), which was similar to those in the LHSM and Chinese Cling genomes, suggesting that SVs specific to these three genomes may regulate genes with similar functions.

#### Long terminal repeat-retrotransposons (LTR-RTs) in TJSM genome

LTR-RTs play an important role in the formation of agronomic traits [21]. In order to use them effectively in breeding, we made a comprehensive analysis of LTR-RTs in TJSM genome. As shown in Fig. 1e, LTR-RTs exhibited a relatively uniform distribution in the TJSM genome. Moreover, the TJSM genome had the larger number of LTR-RTs than the other genomes (Fig. S11, see online supplementary material). These LTR-RTs were classified into five types according to their diversity (%) (Fig. S12, see online supplementary material). The genes with the type I LTR-RT insertions were mainly enriched in the nucleus metabolic process. The genes with the type II LTR-RT insertions were mainly enriched in glycosylation, transportation, and the organic cyclic compound metabolic processes, and these processes occurred in the cytoplasm. The genes with the type III LTR-RT insertions were mainly enriched in metabolic processes associated with the Golgi apparatus. The genes with the type IV LTR-RT insertions were mainly related to pyrophosphatase and hydrolase activity (Fig. S13, see online supplementary material). These results indicated that different types of LTR-RTs might be involved in regulating different functions of genes.

To analyse the evolutionary characteristics of LTR-RTs, we selected 1603 intact LTR-RTs from the five types of LTR-RTs, which ranged from 2000 bp to 7000 bp (Fig. S14, see online supplementary material). Of these LTR-RTs, 1161, 1154, 1199, and 1163 were shared between TJSM and Chinese Cling, Lovell, LHSM, or RYP1, respectively (Fig. S15, see online supplementary material). However, only 67, 17, 15, and 22 were specific to the TJSM genome in comparison with Chinese Cling, Lovell, LHSM, and RYP1 genomes, respectively (Fig. S15, see online supplementary material). This indicated that most of the LTR-RTs might have been inserted in the common ancestor of these five peach cultivars, while only a small proportion of LTR-RTs were inserted after their divergence. In addition, most of the shared LTR-RTs were inserted 2 million years ago (Fig. S16, see online supplementary material), suggesting that the surviving LTR-RTs were selectively neutral or mildly deleterious to their peach hosts and were adequate to be used for tracking the peach origin and evolution process.

### Section 2: identification and analyses of key variations and LTR-RTs responsible for blood-flesh trait

#### Identification of variations and LTR-RTs from anthocyanin biosynthetic pathway genes

The blood flesh is a major agronomic trait of the TJSM variety, and after the TJSM genome assembly and analyses were completed in the beginning of 2019, the key variation for its formation has not been identified (the key variant of blood-flesh trait was reported by Hara-Kitagawa *et al.* in 2020) [27]. Considering this, we mainly investigated the genes in the anthocyanin biosynthetic pathway, which carried the specific SNPs, indels and SVs. As shown in Tables S12 andS13 (see online supplementary material), two genes *Prupe.5G203600* and *Prupe.1G003000* were found to own SNPs and indels specific to TJSM genome. The *Prupe.5G203600* encoded chalcone synthase (CHS) and *Prupe.5G203600* encoded flavonoid 3′-monooxygenase (F3′H), both of which are main enzyme in anthocyanin biosynthesis. Comparison of genome resequencing data showed that specific SNPs and indels on *Prupe.5G203600* were not detected in many blood-flesh varieties, while variations on *Prupe.1G003000* were detected in almost all blood-flesh varieties (Figs S17–S20, see online supplementary material). This indicated that SNPs and indels on *Prupe.1G003000* may be the main variations for blood-flesh formation of the TJSM variety. Since these variations on *Prupe.1G003000* were identified on its promoter, we detected the expression level of this gene in blood-flesh varieties carrying these variations mentioned above. We found that *Prupe.1G003000* was highly expressed in some blood-flesh varieties, while it was low in others (Table S14, see online supplementary material), indicating that these variations were not the key variations for *Prupe.1G003000* expression, and may play a limited role in blood-flesh formation of the TJSM variety.

Two genes *Prupe.5G203600* and *Prupe.2G296600* were found to own specific SVs of TJSM (Fig. 1f). The *Prupe.5G203600* encoded flavonoid 3′-monooxygenase (F3′H) [22], harbors a 236 bp insertion at the end of 3′UTR. The *Prupe.2G296600* encodes cytochrome monooxygenase P450 [23] and carries a 171 bp deletion on its exon. According to the genetic variation location in genes, the 171 bp deletion might have greater effect on gene function than the 236 bp insertion. Currently, two genes *Prupe.2G296600* and *Prupe.1G348000* encoding cytochrome monooxygenase P450 have been reported to be highly expressed in the ripening fruit of TJSM variety [23]. In our study, we found that the expression level of *Prupe.2G296000* was significantly higher than *Prupe.1G348000* in blood and non-blood-flesh peach fruits (Table S14, see online supplementary material), indicating that *Prupe.2G296000* was the main P450 synthesis gene. However, the 171 bp deletion in *Prupe.2G296000* was not observed in most of the blood-flesh varieties (Fig. S21, see online supplementary material), suggesting that this variation may not be the key variation for blood-flesh formation of the TJSM variety.

Among these genes with LTR-RTs specific to the TJSM genome, two genes *Prupe.5G006200* and *Prupe.1G512000* were related to the anthocyanin biosynthetic pathway (Fig. 1f). *Prupe.5G006200* harbored a 6688 bp LTR-RT at 653 bp upstream from ATG. *Prupe.1G512000* encoded acetyl-coAcarboxylase (ACC) and carried an 812 bp LTR-RT at upstream ATG. The *Prupe.1G512000* showed almost the same level of expression in TJSM, Chinese Cling, and RYP1 varieties, suggesting that the 812 bp LTR-RT had little effect on *Prupe.1G512000* expression (Table S14, see online supplementary material). The 6688 bp LTR-RT was detected in all blood-flesh varieties, and *prupe.5G006200* carrying this variation showed high level of expression in all varieties (Fig. 3a; Supplementary Data Table S16). In addition, *Prupe.5G006200*, designated as *PpBL* in a previous study, is a key transcription factor responsible for blood-flesh formation [24]. Thus, the 6688 bp LTR-RT (referred to as blood TE) on the promoter of *Prupe.5G006200* might be the key variation for blood-flesh formation of TJSM variety.

#### A 6688 bp LTR-RT (named blood TE) as the key factor determining peach blood-flesh formation

The blood flesh of peach fruit is classified into two types according to anthocyanin accumulation time [22]. In type I, the anthocyanin content peaks during the ripened fruit stages. In type II, the anthocyanin content reaches its highest level during the un-ripened fruit stages. Type I blood flesh is determined by a NAC family gene *PpBL* located at the top of chromosome 5 [24]. *PpBL* up-regulates the transcription level of *PpMYB10.1*, further promoting the expression of anthocyanin biosynthesis genes, eventually resulting in anthocyanin accumulation in peach fruit [25]. Type II blood flesh is determined by a recessive gene (*bf*) located on chromosome 4 [26].

The blood flesh of TJSM is type I, and anthocyanin began to accumulate in large quantities approximately10 days before fruit ripening. Moreover, the fruit flesh turns dark purple when the fruit is fully ripened. To further determine whether the blood-flesh trait is also controlled by *PpBL* in TJSM variety, we used BSA (bulk segregant analysis) to localize blood-flesh trait. The results showed that there was an obvious peak on top of chromosome 5, and the peak was near the region where *PpBL* was located in the previous study [24] (Fig. S22, see online supplementary material), indicating that *PpBL* was the major gene involved in the blood-flesh formation of TJSM variety. *PpBL* is characterized by high expression levels in the late stage of blood-fleshed fruit development [24]. However, variation leading to the high expression of *PpBL* has not been fully elucidated.

**Figure 2:**
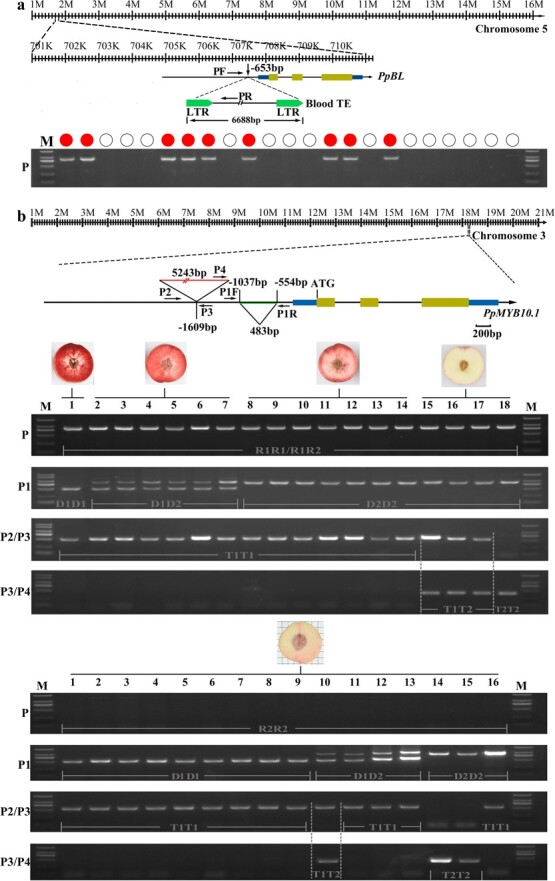
Correlation between blood TE and blood-flesh color in peach. **a** Correlation between genotype of blood TE and phenotype of fruit flesh color in 20 cultivated varieties and 333 individuals derived from two F_1_ populations (Table S15, see online supplementary material). M indicates DNA marker. The varieties with gel bands displayed blood-flesh color fruits, the varieties without gel bands presented non-blood-flesh color fruits.The presence of gel bands amplified using primers PF/PR represent the blood TE on *PpBL* promoter. **b** Correspondence between genotypes of three variations and different intensities of blood-flesh color in cultivated varieties. R1 and R2 represent haplotypes with or without blood TE insertion, respectively. Long and short gel bands amplified using primers P1F/P1R represent absence and presence of the deletion sequence on the promoter of *PpMYB10.1*, respectively, with the long band corresponding to the D_2_ haplotype and the short band corresponding to the D_1_ haplotype. The gel bands amplified using primers P2/P3 and P3/P4 indicates absence and presence of the 5243 bp transposon, respectively, with the band from P2/P3 corresponding to the T_1_ haplotype and the band from P3/P4 corresponding to the T_2_ haplotype. The numbers from 1 to 18 and 1 to 16 indicate different varieties (Table S16, see online supplementary material).

We found that the blood TE was present in all 143 blood-flesh individuals of two F_1_ populations (‘Yangzhou431’ × ’Zhong 07-4Xi-28’ and ‘Zhao Hui’ × ’Zhong 09-1Xi-28’), but it was absent in all 190 non-blood-flesh individuals (Fig. 2a; Table S15, see online supplementary material). Similarly, the blood TE was present in cultivated varieties with blood-flesh fruit, but it was almost absent in non-blood-flesh cultivated varieties (Fig. 2a; Table S15, see online supplementary material), which was consistent with the report by Hara-Kitagawa *et al.* [27]. The results indicated that blood TE was a major factor responsible for the blood-flesh trait.

Thereafter, we found that the blood TE was not only associated with the blood-flesh trait, but also interacted with two variants (a transposon and a deletion mutant) to determine the intensity of fruit blood-flesh color. These two variants are present on the promoter of *PpMYB10.1*, an important gene regulating anthocyanin accumulation located on chromosome 3 [25]. The transposon (5243 bp) is inserted at a position 1610 bp distant from the start codon of *PpMYB10.1* (Fig. 2b). The 483 bp deletion mutant appears at a position 554 bp upstream from the start codon (Fig. 2b).

To reveal the relationship of these two variants and blood TE in blood-flesh formation, two agarose gel bands of different sizes were amplified in the deletion locus using primers P1F/P1R. The short gel band, designated as D_1_ haplotype, had the deletion sequence, and the long gel band, named D_2_ haplotype, did not contain the 483 bp deletion sequence. Another two agarose gel bands, named T_1_ and T_2_ haplotypes, were amplified at the transposon insertion locus using primers P2/P3 and P3/P4, respectively. The results showed that in plant materials with blood TE on *PpBL* promoter, the variety with homozygous genotypes D_1_D_1_ and T_1_T_1_ presented dark blood-flesh color; the varieties with both heterozygous D_1_D_2_ and homozygous T_1_T_1_ genotypes presented blood-flesh color; the varieties with homozygous genotypes D_2_D_2_ and T_1_T_1_ displayed light blood-flesh color; and the varieties with homozygous D_2_D_2_ and heterozygous T_1_T_2_ genotypes exhibited no blood-flesh color (Fig. 2b; Table S16, see online supplementary material). However, in plant materials with no blood TE on *PpBL* promoter, variety carrying any one of the abovementioned genotype displayed non-blood-flesh color (Fig. 2b; Table S16, see online supplementary material). Notably, the above relationship between fruit colors and genotypes was only observed when the blood TE was present on the *PpBL* promoter. All these results suggested that the blood TE was a factor determining blood and non-blood-flesh colors, and that only in the presence of blood TE could the 483 bp deletion and 5243 bp transposon play a role in determining different intensities of blood-flesh color.

**Figure 3:**
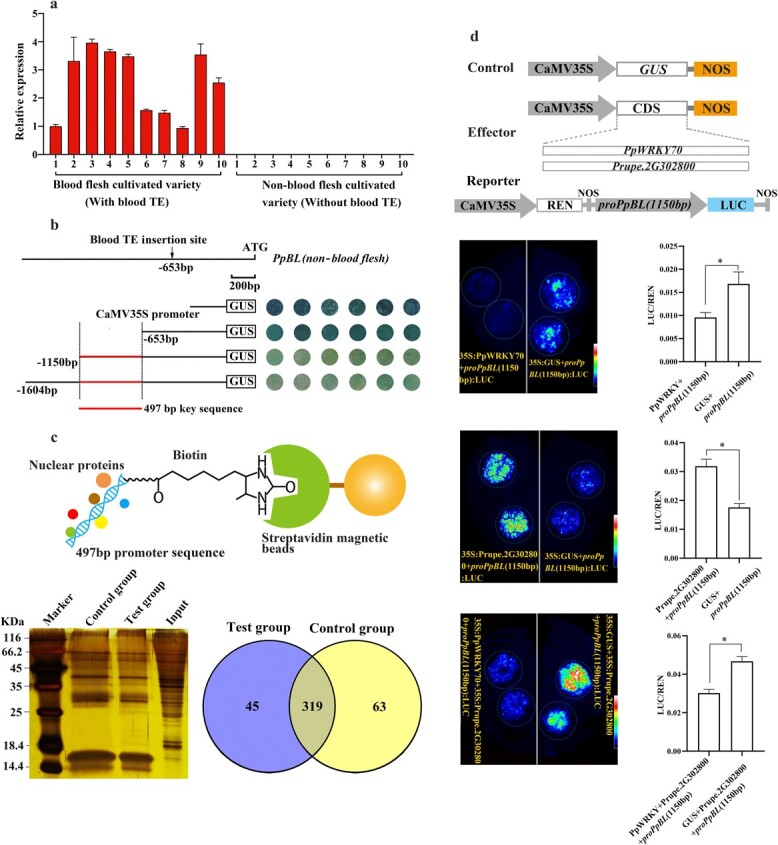
Effect of blood TE insertion on *PpBL* expression. **a** Relative expression levels of *PpBL* in blood-flesh and non-blood-flesh cultivated varieties. The numbers from 1 to 10 indicate blood and non-blood varieties (Table S23, see online supplementary material). **b** Activity detection of *PpBL* promoter with different lengths of sequences in transiently transfected peach fruit discs. Black lines indicate different length promoter sequence of *PpBL*. CAMV35S promoter is used as a positive control. **c** Candidate genes identified by DNA pull-down assay. Input indicates total protein extract. The numbers 45 and 63 are the number of nuclear proteins identified by the test group and the control group, respectively. The number 386 is the number of nuclear proteins shared by the test group and the control group. **d** Regulatory relationship of PpWRKY70 and *PpBL* promoter in transiently transfected *Nicotiana benthamiana* leaves. 35S and NOS indicate CaMV35S promoter and NOS terminator, respectively. ‘*’ indicates significant differences at P < 0.05.

**Table 2:** Candidate genes identified by DNA-pull down assays.

NCBI accession no.	GDR accession no.	Description
A0A251NDN9	Prupe. 7G191000	1-phosphatidylinositol-3-phosphate 5-kinase
A0A251QUL3	Prupe. 1G088100	CASP-like protein
A0A251RJD2	Prupe. 1G566800	Uncharacterized protein
A0A251QDV8	Prupe. 2G101800	Glycosyltransferase
M5XAR3	Prupe. 1G573400	Transmembrane 9 superfamily member
A0A251NP16	Prupe. 6G069100	Uncharacterized protein
M5WMI1	Prupe. 5G208200	UDP-arabinopyranose mutase
A0A251Q3C4	Prupe. 3G204500	Uncharacterized protein
M5X1G1	Prupe. 1G076300	Uncharacterized protein
A0A251QUJ4	Prupe. 1G091000	Uncharacterized protein
A0A251MX91	Prupe. 8G083300	Uncharacterized protein
A0A251QG30	Prupe. 2G147600	ANK_REP_REGION domain-containing protein
A0A251NC00	Prupe. 7G156000	AAA domain-containing protein
A0A251N0I0	Prupe. 8G199600	RING-type domain-containing protein
M5W3L9	Prupe. 7G152400	Uncharacterized protein
A0A251R2D6	Prupe. 1G217100	Uncharacterized protein
M5WHT5	Prupe. 4G040800	Carboxypeptidase
A0A251PL07	Prupe. 4G152400	Uncharacterized protein
A0A251QCW9	Prupe. 2G078200	RNA helicase
A0A251RGB7	Prupe. 1G515900	Protein kinase domain-containing protein
A0A251QMH5	Prupe. 2G275300	Uncharacterized protein
A0A251MYP2	Prupe. 8G159900	Urb2 domain-containing protein
M5XW03	Prupe. 1G422000	Exocyst subunit Exo70 family protein
A0A251MUG3	Prupe. 8G071800	SCP domain-containing protein
A0A251PH10	Prupe. 4G073200	Uncharacterized protein
**M5XHJ0**	**Prupe. 2G265000**	**Probable WRKY transcription factor 70**
A0A251MT84	Prupe. 8G046900	Laccase
M5X7S8	Prupe. 4G070000	MACPF domain-containing protein
A0A251QSC0	Prupe. 1G042000	Uncharacterized protein
A0A251QB81	Prupe. 2G048300	UEV domain-containing protein
A0A251MRD1	Prupe. 8G017900	Uncharacterized protein
M5XY55	Prupe. 1G057000	S-protein homolog
A0A251RER5	Prupe. 1G484500	Uncharacterized protein
A0A251P6U7	Prupe. 5G113200	Tr-type G domain-containing protein
M5XCN6	Prupe. 1G587900	Endoglucanase
M5XTM9	Prupe. 1G503300	Ubiquitin-fold modifier 1
M5X5F5	Prupe. 4G150600	UBC core domain-containing protein
A0A251R7W7	Prupe. 1G347400	Uncharacterized protein
M5XRH3	Prupe. 2G102100	DUF1336 domain-containing protein
A0A251R0X5	Prupe. 1G208100	Uncharacterized protein
A0A251REI5	Prupe. 1G480900	Uncharacterized protein
M5WPL0	Prupe. 5G062600	Lipase_3 domain-containing protein
A0A251RCH1	Prupe. 1G443200	RNA_pol_sigma70 domain-containing protein
A0A251NLC5	Prupe. 6G013400	Peroxidase
M5VQK6	Prupe. 7G257400	DDE Tnp4 domain-containing protein

#### Blood TE as an enhancer of *PpBL* expression

We analysed the expression of *PpBL* in individuals with and without blood-fleshed fruit and found that this gene was highly expressed only in the cultivated varieties carrying blood TE, suggesting that the blood TE insertion changed the expression of *PpBL* (Fig. 3a). It has been reported that LTR can up-regulate the expression of its flanking genes [28]. Therefore, we first investigated the activity of a 1169 bp promoter sequence, composed of the 516 bp LTR of the bloodTE and the 653 bp sequence, using transient promoter-GUS assay in peach fruit. The results showed that the promoters with or without the 516 bp LTR had similar activity to the GUS gene (Fig. S23, see online supplementary material), suggesting that the LTR had little effect on *PpBL* expression. Afterwards, three different promoter sequences of *PpBL* were selected for a transient transformation assay in peach fruit, which is characterized by yellow flesh color, late fruit maturity date, and no blood TE insertion. The results showed that the short sequence (653 bp) from ATG to the blood TE insertion position exhibited a high activity to the GUS gene. On the contrary, two long sequences (1150 bp and 1604 bp) all contained the 653 bp short sequence, showed weak ability to activate *GUS* expression (Fig. 3b). These results indicated that the low-activity region of the *PpBL* promoter was the 497 bp sequence (from −653 bp to −1150 bp). We speculated that this 497 bp sequence might contain *cis*-regulatory elements bound to a transcription suppressor, thus leading to a decrease in *PpBL* expression.

To verify this speculation, the 497 bp promoter sequence was designed as a probe, labeled with a biotin, and incubated with peach fruit nucleoproteins to identify the transcription suppressors binding to this sequence (Fig. 3c). As a result, 45 genes were identified (Table 2), of which a WARK family gene was designated as *PpWRKY70* (also known as *Prupe.2G265000*) (Fig. S24, see online supplementary material) and selected for subsequent analysis. Dual luciferase assay showed that PpWRKY70 displayed a weaker ability to activate the 1150 bp sequence of *PpBL* promoter in infiltration sites of tobacco leaves than the control (Fig. 3d). *Prupe.2G302800* was a gene identified in other DNA pull-down assays with strong luciferase activity to *PpMYB10.1* promoter in separate tobacco leaf infiltration [29]. In the present study, infiltration of Prupe.2G302800 could activate the 1150 bp sequence of the *PpBL* promoter. However, when tobacco leaves were co-infiltrated with Prupe.2G302800 and PpWRKY70, a weak luciferase activity was observed in the infiltration sites compared with the control ‘Prupe.2G302800 + GUS’ (Fig. 3d). All these above-mentioned results suggested that PpWRKY70 might be a negative transcription regulator to *PpBL.* In addition, the luciferase activity at sites infiltrated with 35S:PpWRKY70 + *proPpBL(1150 bp)*:LUC was weaker than that at sites infiltrated with 35S:PpWRKY70 + *proPpBL(653 bp)*:LUC, suggesting that PpWRKY70 might down-regulate *PpBL* transcription by binding to the 497 bp sequence (from 653 bp to 1150 bp) (Fig. S25, see online supplementary material).

### Section 3: effect of key blood-flesh LTR-RT (blood TE) on fruit maturity date (MD)

#### Association of blood TE with advancement of peach fruit MD

We investigated the corresponding relationship between bloodTE and blood-flesh traits, and found that in the two F_1_ populations, a large proportion of blood-fleshed individuals carrying blood TE had virtually no 120–150 days of fruit development period (FDP) (Table S17, see online supplementary material), indicating that blood TE was closely related to the maturity date (MD) of blood-fleshed fruits. Currently, there are two known QTLs controlling the MD of peach fruit, one of which is located on chromosome 4, and the other on chromosome 6 [30–31]. However, neither of these two QTLs is located on the same chromosome with blood TE, as *PpBL* carrying blood TE is located on top of chromosome 5 [24]. This indicated that there was no linkage relationship between MD and blood TE.

In the present study, the QTL located on chromosome 4 had a pronounced effect value (71.3% and 41.4%) on the MD in blood and non-blood-flesh individuals of F_1_ populations (Table S18, see online supplementary material). However, the effect value of QTL on chromosome 6 on the MD was <2% in blood and non-blood-flesh individuals of F_1_ populations, which was significantly lower than the effect value of 20% reported in previous studies [30], indicating that the MD in both populations was mainly controlled by the QTL on chromosome 4 (Table S18, see online supplementary material).

Pirona *et al.* have identified a candidate gene *Prupe.4G186800* at the QTL location on chromosome 4 [32]. The *Prupe.4G186800* had a 9 bp insertion in the third exon, and the 9 bp insertion is co-segregated with the MD in two F_1_ populations with the early ripening individuals harboring the 9 bp insertion and the late ripening individuals carrying no 9 bp insertion (hereafter referred to as carrying 0 bp insertion) [32]. This association between MD and 9 bp insertion is consistent with our analysis results of 64 varieties that 60–90, 90–120, and 120–150 days of FDP was associated with the 9 bp/9 bp, 0 bp/9 bp, and 0 bp/0 bp genotypes, respectively (Tables S19 andS20, see online supplementary material).

F_1_ population (derived from the cross ‘Yangzhou 431’ (0 bp/9 bp) × ’Zhong07-4Xi-28’ (0 bp/9 bp)) without the blood TE included nine individuals with an FDP of 60–90 days, 31 individuals with an FDP of 90–120 days, and 19 individuals with an FDP of 120–150 days. The ratio of the individuals (9:31:19) was statistically in accordance with Mendelian segregation ratio of 1(9 bp/9 bp):2(0 bp/9 bp):1(0 bp/0 bp). In this population carrying the blood TE, 30, 20, and 0 individuals had FDPs of 60–90, 90–120, and 120–150 days, respectively, with the ratios of the individuals being not in accordance with 1(9 bp/9 bp):2(0 bp/9 bp):1(0 bp/0 bp) (Table 3). Similarly, in another F_1_ population (derived from the cross ‘Zhao Hui’ (0 bp/0 bp) × ’Zhong 09-1Xi-28’ (0 bp/9 bp)) carrying the blood-TE, the ratio of individuals with the FDPs of 90–120 days and 120–150 days did not conform with the Mendelian segregation ratio of 1(0 bp/9 bp):1(0 bp/0 bp). However, in this F1 population carrying no blood TE, the ratio of individuals with the two types of FDPs was close to the ratio of 1(0 bp/9 bp):1(0 bp/0 bp) (Table 3). The results indicated that in individuals with blood TE, the actual segregation ratio of maturity date was not consistent with the theoretical segregation ratio of its major gene.

**Table 3:** Segregation of maturity date (MD) in two F_1_ populations crossed from blood and non-blood-flesh varieties.

F_1_ segregating populations		Fruit development period (FDP days)			Expectation ratio	χ^2^	χ^2^_0.05_
		60–90	90–120	120–150			
‘Yangzhou431’ (0 bp/9 bp) × ’Zhong07-4xi-28’(0 bp/9 bp)	Individual without blood TE	**9**	**31**	**19**	1:2:1	3.54	5.99
	Individual with blood TE	**36**	**20**	**0**	1:2:1	50.86	5.99
‘Zhaohui’(0 bp/0 bp) × ’Zhong09-1xi-28’ (0 bp/9 bp)	Individual without blood TE	6	**50**	**75**	1:1	4.39	3.84
	Individual with blood TE	32	**54**	**1**	1:1	31.08	3.84

**Figure 4:**
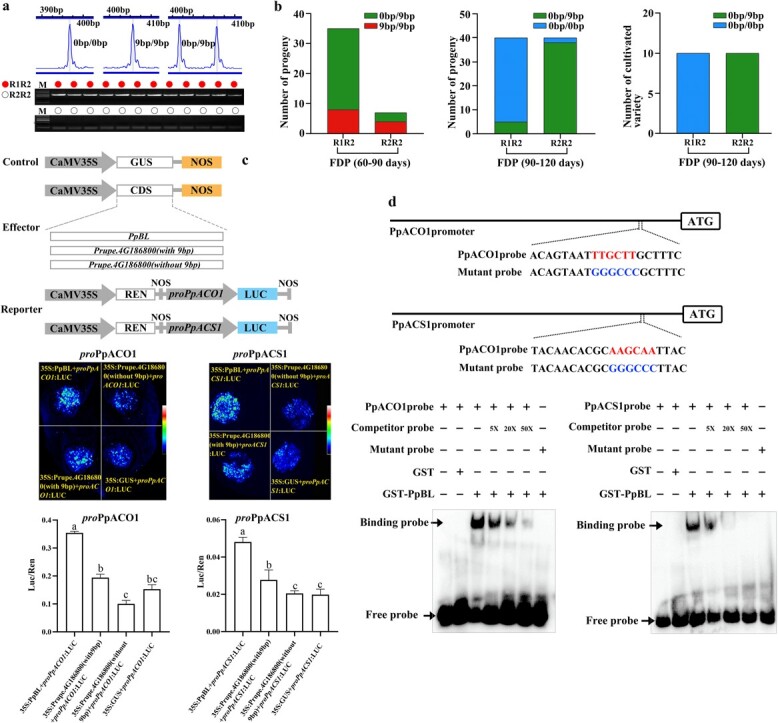
Relationship between blood TE and fruit maturity date. **a** Identification of the 9 bp insertion in the last exon of *Prupe.4G186800* and blood TE on promoter of *PpBL.* The peak between 390 bp–400 bp indicates 0 bp/0 bp genotype. The peak between 400 bp–410 bp indicates 9 bp/9 bp genotype. Two peaks simultaneously occurring at 390 bp–400 bp and 400 bp–410 bp denotes 0 bp/9 bp genotype. R1 and R2 represent haplotypes with or without blood TE insertion, respectively. **b** Correspondence between genotype of 9 bp insertion and blood TE in individuals and cultivated varieties (Table S17, see online supplementary material). **c** Regulatory relationship of PpBL, Prupe.4G186800 (with and without 9 bp insertion), and *proPpACO1*, *proPpACS1* in transiently transfected *Nicotiana benthamiana* leaves. NOS indicates NOS terminator. Different letters indicate significant differences at *P* < 0.05 **d** EMSA showing that PpBL binds directly to the *cis*-acting element of *PpACO1* and *PpACS1* promoter. 5×, 10×, and 50× indicate the relative concentration of competitor probe. ‘+’ and ‘–’ indicate presence and absence, respectively.

To further explore the reason for the above-mentioned inconsistency, 42 and 80 individuals with the FDPs of 60–90 and 90–120 days, respectively, were selected from the two aforementioned F_1_ populations. The 9 bp insertion and blood TE insertion was further identified from these materials through PCR. R_1_ and R_2_ represented haplotypes with or without blood TE insertion, respectively (Fig. 4a). The results showed that the proportion of the R_1_R_2_ genotype of individuals with an FDP of 60–90 days was apparently higher than that of the R_2_R_2_ genotype. Most individuals with R_1_R_2_ harbored the 0 bp/9 bp genotype, whereas individuals with R_2_R_2_ had the same proportions of 9 bp/9 bp and 0 bp/9 bp. For the individuals with an FDP of 90–120 days, most R_1_R_2_ individuals harbored the 0 bp/0 bp genotype, whereas most R_2_R_2_ individuals had the 0 bp/9 bp genotype. For cultivated varieties with an FDP of 90–120 days, all the R_1_R_2_ individuals exhibited the 0 bp/0 bp genotype, whereas the R_2_R_2_ individuals had the 0 bp/9 bp genotype (Fig. 4b; Table S17, see online supplementary material). It could be seen from the above that most individuals with an FDP of 60–90 days and blood TE exhibited the genotype 0 bp/9 bp, and this genotype theoretically should belong to individuals with an FDP of 90–120 days. Similarly, most individuals with an FDP of 90–120 days and blood TE presented the genotype 0 bp/0 bp which theoretically should belong to individuals with an FDP of 120–150 days. These indicated that plants with blood TE displayed the advancement in fruit maturity date, and this advancement might be the major factor resulting in the above-mentioned inconsistency between the actual segregation ratio of maturity date and the theoretical segregation ratio of its major gene.

#### Role of blood TE in fruit maturity advancement

Blood TE insertion is closely related to high levels of *PpBL* expression. PpBL is the transcription factor of the NAC family, which have been reported to be involved in fruit maturity by interacting with ethylene signaling-related genes [33]. Based on this, we speculated that *PpBL* might make contributions to peach fruit maturity. To verify this speculation, the coding sequence (CDS) of *PpBL*, *Prupe.4G186800* (with and without 9 bp insertion) and GUS gene (negative control) were inserted into the pBI121 vector under the control of the CaMV35S promoter, the promoter sequences upstream of the start codon of two peach genes *PpACO1* and *PpACS1* (two rate-limiting enzyme-encoding genes in the ethylene synthesis pathway) were cloned and infused into the pGreenII0800LUC vector. All these recombinant vectors were infiltrated into young leaves of *Nicotiana benthamiana* plants. The results showed that PpBL had more promotor activity towards *PpACO1* and *PpACS1* expression than Prupe.4G186800 (without 9 bp insertion) and GUS (Fig. 4c). This indicated that PpBL might be a positive regulator of ethylene biosynthesis, and the up-regulating effect of PpBL on the expression of *PpACO1* and *PpACS1* was stronger than that of Prupe.4G186800 (without 9 bp insertion). Afterwards, the electrophoretic mobility shift assay (EMSA) demonstrated that PpBL could bind to the *cis*-acting element of *PpACO1* and *PpACS1* promoter *in vitro*, indicating that PpBL activated *PpACO1* and *PpACS1* transcription by directly binding to their promoters (Fig. 4d).

### Section 4 origin of blood TE

In order to explore the origin of the blood TE, a total of 112 landraces and wild varieties were investigated. The results showed that this blood TE was not detected in all wild varieties. Similarly, none of the landraces from the Northwest China group, Yun-gui plateau group (China), Northeast China group, and South China subtropical area group contained the blood TE. In contrast, blood TE was detected in some landraces from the Yangtze River middle and lower reaches group and China North Plain group (Table 4; Table S21, see online supplementary material). This indicated that the blood TE was not derived from the wild germplasms genome.

## Discussion

### Expanded gene family in TJSM genome might be related to its environmental adaptability

Environmental adaptation is important for species survival and biodiversity, especially under the context of major changes in the global environment [34–35]. In China, there are seven major peach landraces groups, and they can reflect local special ecological environment characteristics. Peach accessions have adapted to the local environment by evolving a large number of stress-response genes during a long period of natural selection process [36, 18, 7]. In our study, 78 gene families were found to expand significantly (*P* < 0.05) in the TJSM genome, of which 18 were mainly enriched in the cutin, suberine, and wax biosynthesis pathways. These cutin, suberine, and wax are all secondary metabolites, which were reported to be wildly involved in the physiological processes in response to plant disease, insect pest, and water loss stresses [37–38]. The TJSM variety is from the China North Plain group whose environment is characterized by dry and windy climate in the winter. Based on this, we speculated that the 18 expanded genes might play an important role in preventing water loss from tree branches and facilitating TJSM adaption to the local natural environment.

### In addition to *PpBL*, there might be other genes involved in blood-flesh formation of the TJSM variety

The blood flesh of peach fruit is classified into two types according to anthocyanin accumulation time [22]. Type I blood flesh is determined by a NAC family gene *PpBL* located at the top of chromosome 5. PpBL up-regulates the transcription level of *PpMYB10.1* by forming a dimer with PpNAC1, further promoting the expression of anthocyanin biosynthesis genes, eventually resulting in anthocyanin accumulation in peach fruit [24]. In our study, we found that there was a small peak near the previously reported main peak where *PpBL* was located [24] (Fig. S22, see online supplementary material), indicating that apart from *PpBL*, there might be other genes participating in blood-flesh formation. Therefore, we further investigated the SNP distribution in the region near the small peak (Table S22, see online supplementary material). The results showed that although most of the SNPs were distributed in the intergenic region, their effect on nearby genes could not be ruled out. Therefore, the effects of these SNPs need to be further explored in future experiments.

### Blood TE might weaken the transcriptional repression effect of PpWRKY70 on *PpBL*, leading to the blood-flesh formation

**Figure 5:**
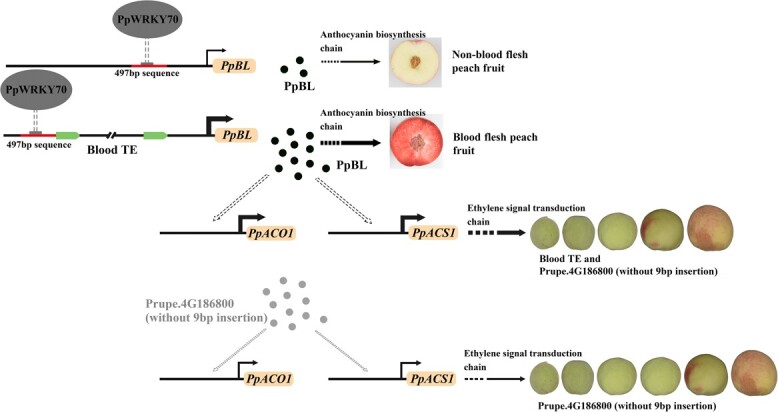
Schematic diagram of proposed model presenting effects of blood TE on peach fruit flesh color and maturity date. The thin and thick lines with arrows indicate low and high level of *PpBL*, *PpACO1*, or *PpACS1* expression, respectively. The black and gray spot indicates the *PpBL* and Prupe.4G186800 (without 9 bp insertion) expression product, respectively. The dotted line pointing to 497 bp sequence indicates PpWRKY70 may down-regulate *PpBL* expression. The thick dotted black arrow pointing to *PpACO1* and *ACS1* indicates that PpBL may strongly up-regulate the expression of *PpACO1* and *ACS1*. The thick dotted arrow pointing to the upside of fruits indicates that a series of upstream reactions may have a strong promoting effect on the maturity of fruit carrying blood TE and *Prupe.4G186800* (without 9 bp insertion). The thin dotted arrow pointing to *PpACO1* and *ACS1* indicates that Prupe.4G186800 (without 9 bp insertion) slightly up-regulates the transcription of *PpACO1* and *ACS1*. A thin dotted arrow pointing to the downside of fruits indicates that a series of upstream reactions have a weak promoting effect on the maturity of fruit carrying *Prupe.4G186800* (without 9 bp insertion).

LTR-RT is an RNA-mediated transposon. The transposition to a new chromosome location needs a copy-and-paste mechanism [39]. Transposons regulate their host gene by many methods, one of which is to act as an insulator to block upstream or downstream gene activation [40]. In our study, when there was no blood TE inserted into the *PpBL* promoter, PpWRKY70 bound to the promoter of *PpBL*, thus significantly repressing its expression. However, when the blood TE existed on the *PpBL* promoter, blood TE separated PpWRKY70 to make it far away from ATG. In this case, *PpBL* showed high expression. The above results indicated that the blood TE insertion might weaken the repression of *PpBL* by PpWRKY70. Based on this, we speculated that PpWRKY70 might repress *PpBL* expression when the *PpBL* promoter carried no blood TE, thus reducing anthocyanin accumulation in the flesh, eventually resulting in a non-blood-flesh phenotype. However, when the blood TE was present on the *PpBL* promoter, this blood TE might act as an insulator to eliminate or attenuate the repression, thus leading to up-regulation of *PpBL* expression and increasing anthocyanin accumulation in the flesh, ultimately leading to a blood-flesh phenotype (Fig. 5).

**Table 4:** Identification of blood TE in landraces and wild peach varieties.

Accessions		Number of accessions	Number of accessions with blood TE	Ratio of the latter to the former (%)
Wild varieties		13	0	0
	Northwest China group	20	0	0
	Yun-gui plateau group	18	0	0
	Northeast China group	9	0	0
	China North Plain group	20	**7**	**35**
	Yangtze River middle and lower reaches group	22	**10**	**45**
Landraces	South China subtropical area group	10	0	0

### Stronger ability of PpBL to promote ethylene synthesis than Prupe.4G186800 (without 9 bp insertion) might be the main factor for maturity date advancement

NAC transcription factors (TFs) usually regulate fruit ripening by modulating the expression of genes in the ethylene pathway. For instance, silencing of *SNAC4* and *SNAC9* will suppress the expression of ethylene pathway-related genes such as *LeACS2*, *LeACS4*, *LeACO1*, and *LeERF2,* thus inhibiting the fruit ripening process [33]. Additionally, silencing of *SlNAC4* by RNA interference (RNAi) decreased the expression of ethylene biosynthesis genes, thus delaying the tomato fruit ripening [41]. NAC TF (NOR-like1) has been reported to have a promoter-binding activity to *SIACS2* and *SIACS4 in vitro* and *in vivo*, and silencing NAC TF can decrease the expression of *SIACS2* and *SIACS4,* thus delaying fruit ripening [42]. In peach fruit, Prupe.4G187100 (a peach NAC) binds to the *PpACO* and *PpACS* promoters and generates autocatalytic ethylene in tobacco transgenic lines [43]. In our study, PpBL, a peach NAC TFs, showed higher promotor activity towards *PpACO1* and *PpACS1* than anther NAC TFs Prupe.4G186800 (without 9 bp insertion), indicating that PpBL might also promote ethylene synthesis and positively regulate fruit ripening, and that the ability of PpBL to promote fruit ripening is higher than that of Prupe. 4G186800 (without 9 bp insertion). Based on this, we speculated that the high expression of *PpBL* and its promoting effect on ethylene synthesis might compensate for the corresponding defects of *Prupe.4G186800* (without 9 bp insertion), thus leading to an earlier maturity date of blood-flesh peach fruit in plants carrying *Prupe.4G186800* (without 9 bp insertion) and the blood TE (Fig. 5).

### Speculation on the origin of blood TE

Retrotransposons are important components of plant genome, and some of them co-evolve with their hosts [40]. In our study, the retrotransposons blood TE was only identified in the landraces originating from the Yangtze River middle and lower reaches group and the China North Plain group, but it was undetected in wild varieties, suggesting the blood TE was not derived from wild varieties genome. Retrotransposon replication is usually restricted by host mechanisms including DNA methylation, histone 3 lysine 9 methylation, and small RNAs [44–47]. However, this restriction can be eliminated under biotic and abiotic stresses such as fungal and bacterial infection, injury, absence of nitrogen, and high temperature [48–51]. Considering that many homologous sequences of the blood TE have been identified among eight chromosomes (Fig. S26, see online supplementary material), we proposed that the blood TE might be replicated from one of these homologous sequences, and this replication might be induced by high temperature and high humidity in Yangtze River middle and lower reach regions.

## Materials and methods

### Plant materials

An ancient, blood-fleshed landrace named Tianjin Shui Mi (TJSM) (Fig. S1, see online supplementary material), was used for assembling a high-quality genome. Two F_1_ cross populations (‘Yangzhou431’ × ’Zhong 07-4Xi-28’ and ‘Zhao Hui’ × ’Zhong 09-1Xi-28’) containing 333 individuals were used for genotype identification of blood TE (Table S15, see online supplementary material). ‘Zhong 07-4Xi-28’ and ‘Zhong 09-1Xi-28’ are blood-flesh progenies of TJSM. ‘Yangzhou431’ and ‘Zhao Hui’ are two non-blood-flesh varieties. Thirty-four cultivated varieties were used to detect the association between genotype of blood TE, 483 bp deletion, 5243 bp insertion, and blood-flesh formation (Table S16, see online supplementary material). Sixty-four varieties were selected to determine the genotype of 9 bp insertion on Prupe.4G186800 (Table S20, see online supplementary material). An F_1_ population (‘Yangzhou 431’ × ’Zhong 07-4Xi-28’) was used to locate the blood-flesh trait. A total of 122 individuals from the two above-mentioned F1 populations were used to determine the relationship between genotype of 9 bp insertion and fruit maturity date (Table S17, see online supplementary material). In total, 112 accessions containing 13 of wild varieties and 99 of landraces were selected to detect the presence or absence of blood TE on *PpBL* promoter (Table S21, see online supplementary material). Twenty blood and non-blood-flesh cultivated varieties were selected to determine the relative expression of *PpBL* in ripened fruit (Table S23, see online supplementary material). All these landraces, wild and cultivated varieties were planted in the national peach germplasm resources repository of Zhengzhou Fruit Research Institute, Chinese Academy of Agricultural Sciences, Zhengzhou, Henan Province, China. All individuals from the two F1 populations were planted in an orchard in Xinxiang city, Henan province, China. All the trees were normally cultivated and managed.

### Genome sequencing and assembly

High-quality genomic DNA was extracted from young leaves of TJSM using the cetyltrimethylammonium bromide method for next-generation sequencing [52]. The extracted DNA was used to construct libraries (350 bp) using the Illumina TruSeq Nano DNA Library Prep kits (Thermo Fisher Scientific, Waltham, MA, USA). Thereafter, the inserted DNA fragments were sequenced on the Illumina HiSeq X Ten platform (Thermo Fisher Scientific). Low-quality and adapter-polluted reads were filtered to obtain high-quality clean reads. The genomic DNA used for the third-generation sequencing was extracted from the same materials by the same method as mentioned above for the next-generation sequencing. High-quality DNA was sheared, purified, and end-repaired to construct the SMRT bell libraries using the SMRT bell Template Prep Kits (Pacific Biosciences, Menlo Park, CA, USA). These library fragments were sequenced using the PacBio Sequel System (Pacific Biosciences). For Hi-C sequencing, young leaves from TJSM were first ground into a powder and fixed with formaldehyde (1% v/v). Then, the crosslink DNA was cleaved into large fragments with restriction enzymes (HindIII and Mbol), and the enzyme-cleaved products were labeled with biotin on both ends of the fragments. Finally, these labeled fragments were ligated, purified, and cleaved into small fragments, and these small fragments were pulled down and sequenced on an Illumina HiSeq platform [53–54]. Different tissues of TJSM variety, including the fruit, leaf, and phloem, were used for transcriptome sequencing to improve gene prediction accuracy. Total RNA was extracted with a kit (Waryong, Beijing, China) and reverse-transcribed with a Supremo III RT kit (BioTeKe, Beijing, China). The mRNA was randomly fragmented with fragmentation buffer and first-strand cDNA was synthesized with random hexamers. Double-stranded cDNA was then synthesized with dNTPs, RNase H, and DNA polymerase I, and enriched by adding poly-(A)s and PCR amplification. The enriched cDNA was linked to a vector and sequenced on the Illumina HiSeq X Ten platform (Thermo Fisher Scientific). Approximately 2.8 Gb of sequencing data from every sample were obtained.

Clean next-generation sequencing data were assembled using SOAP software to evaluate the genome size, heterozygote rate, and repeat percentage of the TJSM genome [55]. Circular consensus sequencing (CCS) reads were assembled using Hifiasm software [56]. The continuity and accuracy of the assembled genome were evaluated using the complete BUSCOs (%) [57]. In addition, the Illumina reads were also mapped onto the genome to evaluate the accuracy and continuity. To build pseudo-chromosomes, the clean paired-end reads filtered from the Hi-C sequenced data were mapped onto the assembled genome using the Bowtie2 and HIC-Pro software. The contigs were clustered, ordered, and oriented using LACHESIS [58–59].

### Genome annotation

Genome annotation was performed in the following four steps, namely, repeat sequence prediction, gene structure prediction, gene function annotation, and non-coding RNA prediction. Rep Base, Repeat Masker, and Repeat protein mask were used to predict these sequences similar to known repeat sequences [60–61]. In addition, Repeat Modeler (http://www.repeatmasker.org/RepeatModeler/) was employed for repeat sequence prediction. Three strategies were adopted for gene structure prediction: Strategy1: transcriptional data were used to predict gene structure using software PASA [62]; Strategy2: a database (http://blast.ncbi.nlm.nih.gov/Blast.cgi) and software Genewise were used to predict the gene structure which was homologous to known coding protein sequences [63]; and Strategy3: the gene structure was *ab initio* predicted using software such as Augustus, SNAP, and Gene Mark based on the genomic sequence data [64–65]. Gene function was annotated based on protein databases including Swiss-prot, NT, NR, PFAM, eggNOG, GO, and KEGG [66]. Non-coding RNA was predicted based on RNA libraries Rfam and tRNAscan-SE [67–68].

### Gene family clustering analysis

Eight plant species were selected for a comparative genomic analysis, including *M. domestica*, *P. persica*, *F. ananassa*, *A. thaliana*, *V. vinifera*, *O. sativa*, *C. sativus*, and *P. trichocarpa*. The genes in each genome were filtered, and only those genes containing only one transcript per gene and coding more than 50 amino acids were retained for the gene family clustering. Thereafter, protein sequences from the genomes of the nine species (eight species +TJSM) were pairwise aligned (e-value <10^−6^), and based on the aligned results, cluster analysis was conducted using software OrthoMCL [69] to obtain the genes with similar function or structure.

### Expansion and contraction in gene families

First, gene families with more than 200 genes and less than two genes were removed from the genome. Then, expansion and contraction analyses of gene families were performed using CAFE software [70] (http://sourceforge.net/projects/cafehahnlab) with the parameters set as *P* < 0.05 and r < 10 000. Gene families expanded and contracted were extracted from the genome of a specific species with CAFE, followed by statistical analysis. Finally, the significantly expanded and contracted gene families were annotated based on GO and KEGG databases.

### Sequence variation identification from four peach variety genomes

For the identification of SNPs and small indels (<50 bp), we aligned four peach varieties genomes (TJSM, Chinese Cling, LHSM, and RYP1) to the reference genome Lovell using mummer 4.0, and then filtered the alignment results using delta-filter with the custom parameters (−1 -i 0.8 -l 500). Finally, we extracted the information on SNPs and indels using show-snps with the custom parameters (−l -r -T). Likewise, four genomes were mapped on the Lovell genome using mummer 4.0. After the low-quality sequences were filtered with delta-filter (−1 -i 0.8 -l 500 parameters), SVs were identified using Assemblytics with the custom parameters (500 5 010 000). Minimap2 and Syri were used to conduct the genome alignment and INV identification with the custom parameters (−ax asm5–eqx) and (−k -F S), respectively. The genes with SV distributions were annotated based on GO databases.

### Analysis of LTR-RTs

LTR_Finder (v1.05) and LTR_Harvest (Genome Tools v1.5.1) [71] were used to identify LTR-RTs from the five peach cultivar genomes (TJSM, Chinese Cling, LHSM, RYP1, and Lovell) with the custom parameters (-D 20000 -d 1000 -L 7000 -l 100 -p 20 -C -M 0.85) and (−similar 85 -vic 10 -seed 20 -seqids yes -minlenltr 100 -maxlenltr 20 000 -mintsd). A total of 1604 intact LTR-RTs were extracted from the outputs of the aforementioned TJSM genome, using the LTR retriever [72]. Both 500 bp fragments upstream and downstream of each intact LTR-RT were first extracted from LTR-RT libraries and then aligned to the TJSM genome using BWA-MEM (parameter: -a) to obtain unique LTR-RTs. The corresponding LTR-RTs of those fragments uniquely aligned to the 20 kb region on the same contig were retained with 51 LTR-RTs removed. Both 500 bp fragments upstream and downstream of these retained LTR-RTs were aligned to the Chinese Cling, Lovell, LHSM, and RYP1 genomes with parameter: -a, and after alignment, 1407, 1413, 1391, and 1408 LTR-RTs for each genome were retained, respectively. The middle sequences between upstream and downstream of LTR-RTs in Chinese Cling, Lovell, LHSM, and RYP1 genomes were aligned to the TJSM genome using blastn with parameter: -F, and the aligned LTR-RTs were classified into five types, namely, high shared, low shared, insertion, elimination, and unknown [21].

### DNA pull-down assay

To identify transcription suppressors binding to 497 bp promoter sequence of *PpBL*, the sequence was synthesized by Henan Write gene Biotechnology CD, Ltd (Henan, China). Fruit nuclear proteins were extracted using a nuclear and cytoplasmic protein extraction kit (Sangon Biotech, Shanghai, China). The oligonucleotide was mixed with streptavidin-magnetic beads and 500 μl incubation buffer and then incubated for 1 h. The DNA-bead complex was isolated and washed two times with nucleic acid and protein incubation buffer. One μg of the DNA-bead complex was incubated with 300 μl of nuclear proteins for 20 min at room temperature. The protein-DNA-streptavidin-agarose complex was analysed with sodium dodecyl sulfate-polyacrylamide gel electrophoresis.

Enzymolysis of gel-containing complex was performed in the following steps to obtain target protein: (i) protein gel fragment was excised using a clean scalpel and washed twice with 200 μl ddH_2_O for 10 min; (ii) 200 μl of destaining solution (50 mM NH_4_HCO_3_ and ACN) was added for fading for 15 min three times until the gel was faded completely; (iii) 200 μl 10 mM DTT was added to the gel and incubated at 37°C for 1 h; (iv) 200 μl 55 mM IAM was added to the gel and incubated in the dark for 30 min; (v) the gel was washed twice, respectively, with ddH_2_O and CAN for 10 min each time; (vi) 100 μl of Trypsin working solution (0.01 μg/μl) was mixed with the gel, and stood at 4°C for 30 min. After the enzyme was completely absorbed by the gel, 100 μl of NH_4_HCO_3_ (25 mM) was added to the gel and incubated at 37°C for 12 h; (vii) the enzymolysis supernatant was collected centrifugally and placed into a new tube. Then, 200 μl of CAN (100%) was added to the remaining gel and vortexed for 5 min, and the enzymolysis supernatant was collected and put into the same tube mentioned above. The collected supernatant was vacuum-dried and stored at −20°C.

The dried enzymolysis supernatant was dissolved in a 0.1% FA solution and centrifuged at 14000 × *g* for 20 min. The supernatant was subjected to mass spectrometry (MS) analysis with the following parameters: (i) ion source: NSI; spray voltage, 2200 V; capillary temperature, 350°C; (ii) full MS: resolution, 120000FWHM; full scan AGC target, 20.e^5^; full scan max.IT, 50 ms; scan range, 250-1450 m/z; (iii) dd-MS2: resolution, 30000FWHM; AGC target, 5.0e^5^; NCE, 30%. The MS data were analysed using Proteome Discoverer 2.4 (Thermo Fisher Scientific).

### Localization of blood-flesh traits based on BSA

To determine whether the blood-flesh trait of TJSM was also controlled by *PpBL*, an F_1_ population crossed from a blood-flesh progeny of TJSM (Zhong 07-4Xi-28) and a non-blood-flesh landrace (Yangzhou 431) was used to locate the blood-flesh trait. Fifteen F_1_ individuals, respectively, with blood flesh and non-blood flesh were selected to construct the mixing pools. The blood-flesh pool was named chi1, and the non-blood-flesh pool was named chi2. The DNA of parents and the mixed pool was extracted using a DNA extraction kit (Aidlab, Beijing, China) and sequenced on the IlluminaHiseqX Ten platform (Thermo Fisher Scientific) at a depth of 30×. The low-quality sequences were removed from raw sequencing data, and the high-quality data were aligned to the reference genome TJSM with Minimap2 [73]. The SNPs were identified and filtered using GATK [74] with filtering parameters of QD < 2.0 || FS > 60.0 || MQ < 40.0 || MQRankSum<−12.5 ||ReadPosRankSum<−8.0 || DP ≥ 4. The SNP-ED of the two mixed pools for each SNP was calculated according to the formula ED = ((Amut−Awt)^2^ + (Cmut− Cwt)^2^ + (Gmut−Gwt)^2^ + (Tmut−Twt)^2^)^0.5^), and each ED value was raised to the power of 5. Then, 100 kb was selected as the sliding window, and 10 kb as the step size. The fifth power value of ED was mapped to the chromosome. The 95% confidence level was set as the screening threshold, and the windows above the 95% confidence level were defined as the candidate interval.

### Validation of two major QTLs controlling maturity date (MD) in two F_1_ populations

Based on the results of previous studies, six and two SNPs closely linked to the two major QTLs of MD were selected on chromosomes 4 and 6 of peach, respectively, and their genotypes were identified in the two F_1_ populations (‘Yangzhou 431’ × ’Zhong 07-4Xi-28’ and ‘Zhao Hui’ × ’Zhong 09-1Xi-28’). The maturity date (MD) was defined based on the fruit development period (FDP), which referred to the number of days from the early full bloom date to the harvest maturity date [75]. SNP genotyping was conducted using Kasp markers. The genotyping results and the MD linkage analysis were performed using JoinMAP4.0 and MapQTL6.0. The SNP position information was listed in an Excel table (Table S24, see online supplementary material). The Kasp primers used in this study were shown in Table S25 (see online supplementary material).

### Transient transformation assay of peach fruit

To identify the activity of different parts of the *PpBL* promoter, three different promoter sequences of it (653 bp, 1150 bp, and 1640 bp upstream the start codon) and one 1169 bp promoter sequence containing a 653 bp and 516 bp LTR of blood TE were cloned and infused into the multiple cloning site (MCS) sequence of pBI121 vector. CaMV35S promoter was used as a positive control [76]. The prepared constructs were transformed into *Agrobacterium tumefaciens* GV3101 and incubated at 28°C for two days. Individual strains were selected from the transformation plate and suspended in 1.0 ml of LB medium containing 50 mg/ml kanamycin. After shaking at 28°C for 10 h, 10 μl of bacterial solution was transferred into 15 ml of LB medium and incubated in a shaking incubator for 8–12 h, and the optical density (OD) was measured and adjusted to 0.4–0.6 using infiltration buffer (containing0.5 M MES, 1.0 mM MgCL_2_, 1.0 μM As). This adjusted bacterial solution was incubated in a non-shaking incubator at 28°C for 2 h and infiltrated into fruit discs using a vacuum centrifugal concentrator (Blonoon, Shanghai, China). Peach fruits approximately 15 days before fruit ripening were used for this transient transformation assay. After two days, the fruit discs were placed in GUS staining buffer (Sangon Biotech) to stain at 37°C for 12–24 h.

### Dual luciferase assay of tobacco leaves

To verify whether TFs had transcriptional activation or inhibitory activities on downstream genes, promoter sequences upstream of the start codon of three peach genes *PpBL, PpACO1*, and *PpACS1* were cloned and infused into the pGreenII0800LUC vector [75–77, 19]. The coding sequence (CDS) of *PpWRKY70*, *Prupe.2G302800*, *PpBL*, and *Prupe.4G186800* (with and without 9 bp insertion) was inserted into the pBI121 vector under the control of the CaMV35S promoter. The GUS gene was inserted into the pBI121 vector as a negative control [24]. All these recombinant vectors were transformed into *A. tumefaciens* GV3101 and incubated at 28°C for two days. Single colonies were selected and incubated in 1.0 ml of LB medium (50 mg/ml kanamycin) with shaking at 28°C for 10 h. Then, 10 μl of the single colonies was transferred into 15 ml of LB medium (50 mg/ml kanamycin) and incubated with shaking at 28°C for 8–12 h, and the OD was measured and adjusted to 0.4–0.6 using infiltration buffer (0.5 M MES, 1.0 mM MgCL_2_, 1.0 μM As). Finally, the bacterial suspension was infiltrated into young leaves of *N. benthamiana* plants. At 2.5 days after the leaf infiltration, leaves were soaked in D-Luciferin sodium salt solution (Sangon Biotech) for 10 min. The luminous pictures of the treated leaves were captured on a multifunctional imaging analysis system (Tanon, Shanghai, China). In addition, the 2.5 days of infiltration leaf was ground into powder to measure the ratio of LUC to Ren activity using Dual-Glo® Luciferase Assay System (Promega) on an Infinite M200 luminometer (Tecan, Mannerdorf, Switzerland).

### Electrophoretic mobility shift assay (EMSA)

EMSA was used to detect whether PpBL could directly bind to the *cis*-acting element of *PpACO1* and *PpACS1* promoter *in vitro*. The CDS sequence of *PpBL* was cloned and inserted into Pgex-6p-1 vector, and the recombinant vector was transformed into Rosetta (DE3) for induction expression. Single-stranded oligonucleotides labeled with or without biotin were used as the probes. The probes were incubated with the nuclear extract at room temperature for 30 min. The entire reaction mixture was run on a non-denaturing 0.5 × TBE 6% polyacrylamide gel for 1 h at 60 V at 4°C and then transferred onto Biodyne® B nylon membranes (Pall Corporation, NY, USA). Signals were visualized with reagents included in the kit and ChemiDoc XRS (Bio-Rad Laboratories, CA, USA) [78].

### Identification of variation sites of *PpBL*, *PpMYB10.1,* and *Prupe.4G186800* in peach segregated populations and cultivated varieties

Genomic DNA was extracted using a DNA extraction kit (Aidlab, Beijing, China). The sequences containing blood TE, 5243 bp transposon, and 483 bp deletion, and 9 bp insert were amplified using primer pairs to obtain PCR products (Table S25, see online supplementary material). PCR products were separated on an agarose gel (m/v = 1%) for identifying the blood TE on the *PpBL* promoter and the 5243 bp transposon and 483 bp deletion on *PpMYB10.1*. The 9 bp insertion on *Prupe.*4G186800 was identified by sequencing the PCR products. The10 μl PCR reaction system contained 5 μl of buffer, 0.4 μl of forward and 0.4 μl reverse primers, 0.2 μl of dNTPs, 0.2 μl of enzyme, and 3.8 μl of ddH_2_O. The PCR program included three steps: (i) pre-denaturation at 95°C for 5 min; (ii) 34 cycles of 95°C for 15 s, 58°C for 15 s, and 72°C for 40 s; and (iii) 72°C for 10 min. The PCR products were stored at 4°C.

### Quantitative real-time PCR

RNA was extracted from fruit flesh using the rapid extraction kit (Aidlab, Beijing, China). Approximately, 1.5 μg of the RNA was reverse-transcribed into cDNA using the Prime Script RT reagent kit equipped with gDNA Eraser (BioTeK, Beijing, China). The 20 μl PCR reaction system contained 2.0 μl cDNA diluted 10 times by adding ddH_2_O, 0.5 μl forward primer, 0.5 μl reverse primer, 10 μl SYBR premix, and 7 μl ddH_2_O. The amplification program was as follows: pre-denaturation at 95°C for 30 s, 45 cycles of 95°C for 15 s, 58°C for 15 s, and finally 72°C for 15 s. A peach *TEF_2_* gene was selected as a control [79].Three technical repeats were assessed.

## Supplementary Material

Web_Material_uhad265
