# Supplementary material for: Genome variation and LTR-RT analyses of an ancient peach landrace reveal mechanism of blood-flesh fruit color formation and fruit maturity date advancement
Source: Hortic Res. 2023 Dec 19;11(1):uhad265. doi: 10.1093/hr/uhad265 (PMC10828781; doi:10.1093/hr/uhad265)
Supplement: Web_Material_uhad265 [file web_material_uhad265.zip › Supllementary Data Figure.docx]

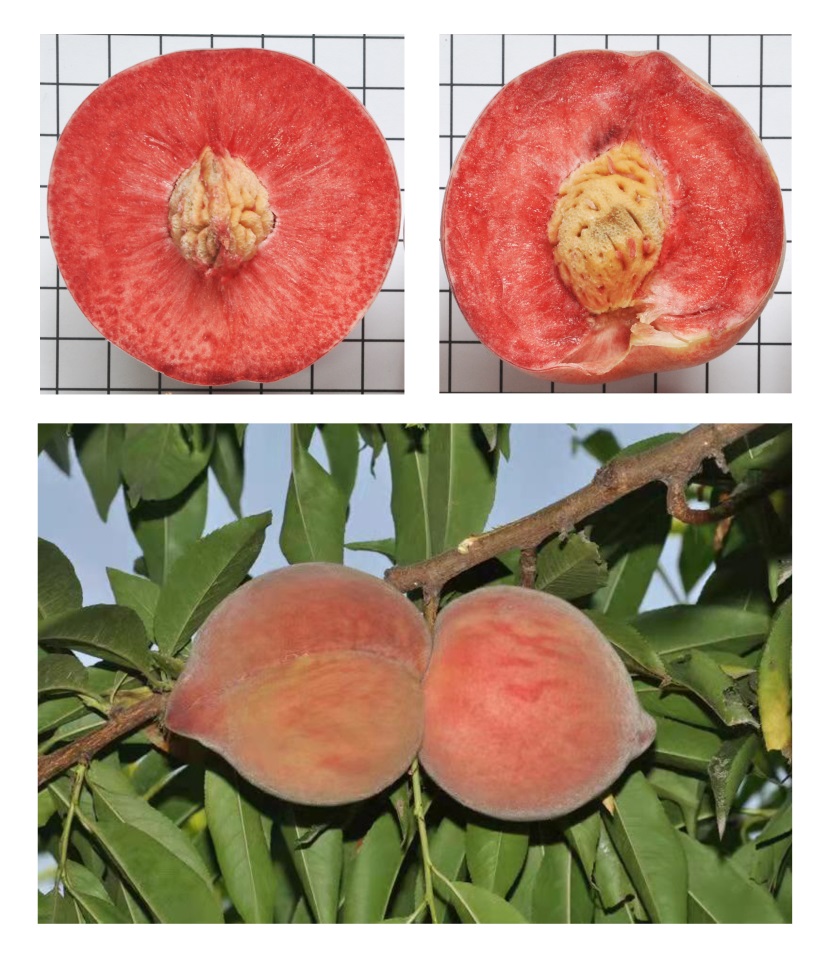


Figure S1 Blood-flesh fruit of a Chinese landrace TJSM


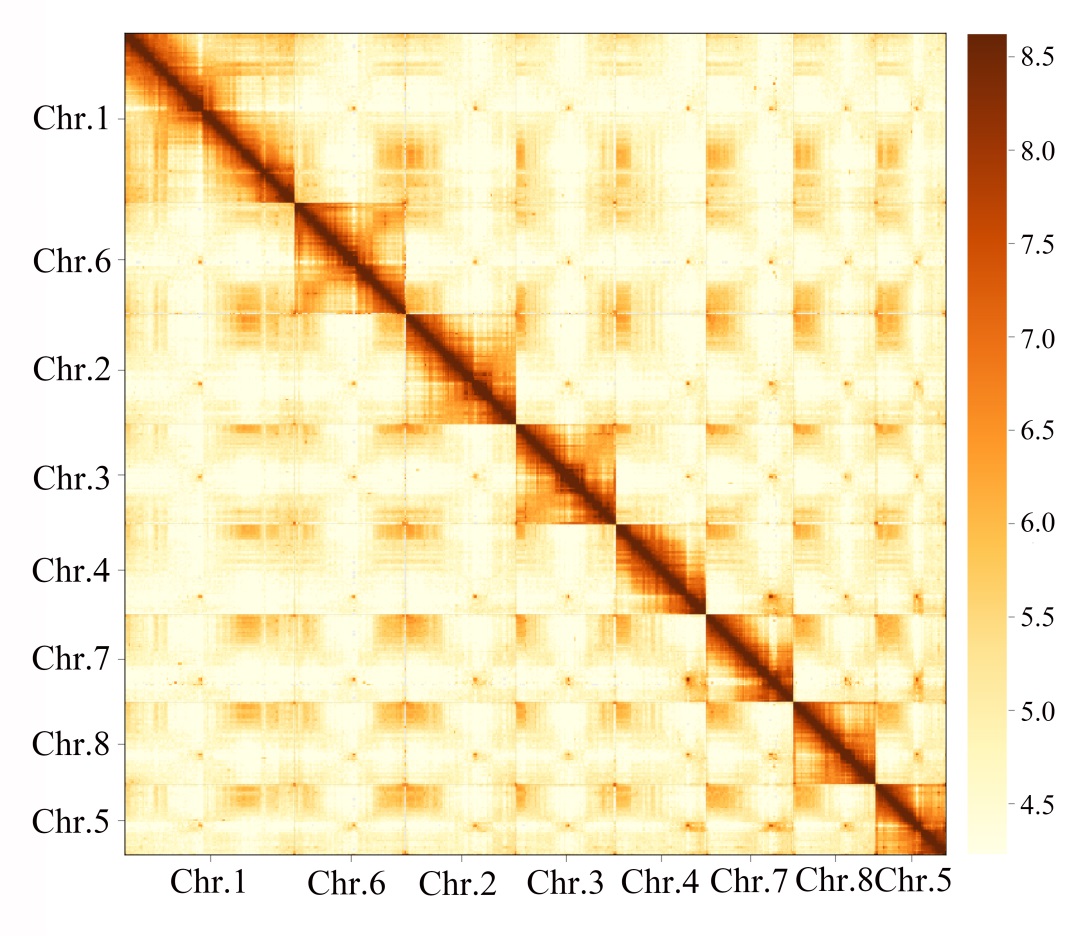


Figure S2 Hi-C interaction heatmap of bins obtained from chromosome of TJSM genome


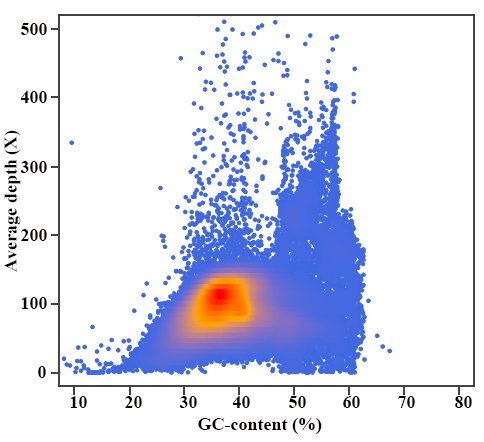


Figure S3 GC content and sequencing depth scatter distribution diagram


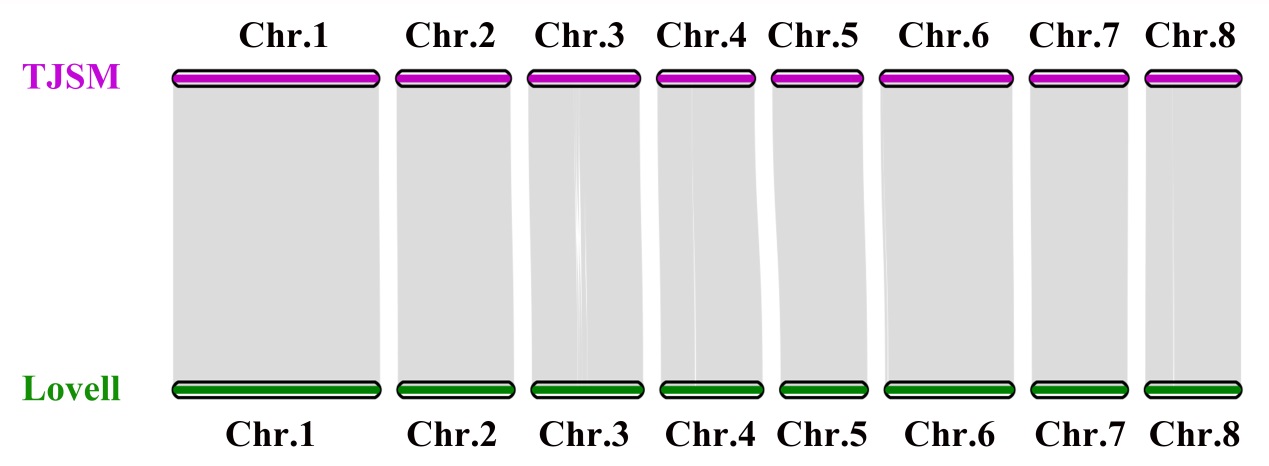


Figure S4 Collinearity analysis between TJSM and Lovell genome


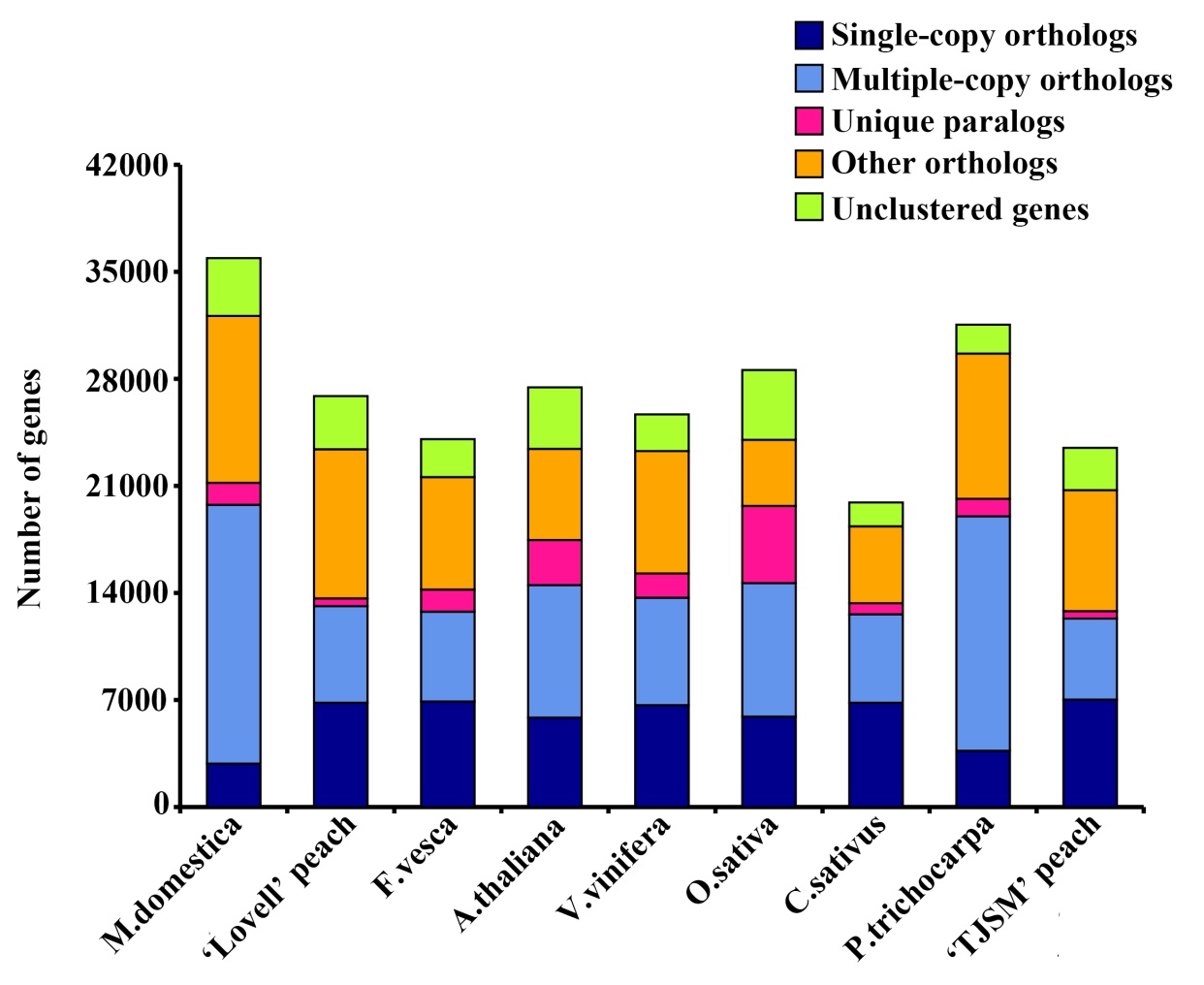


Figure S5 Different types of genes in eight species


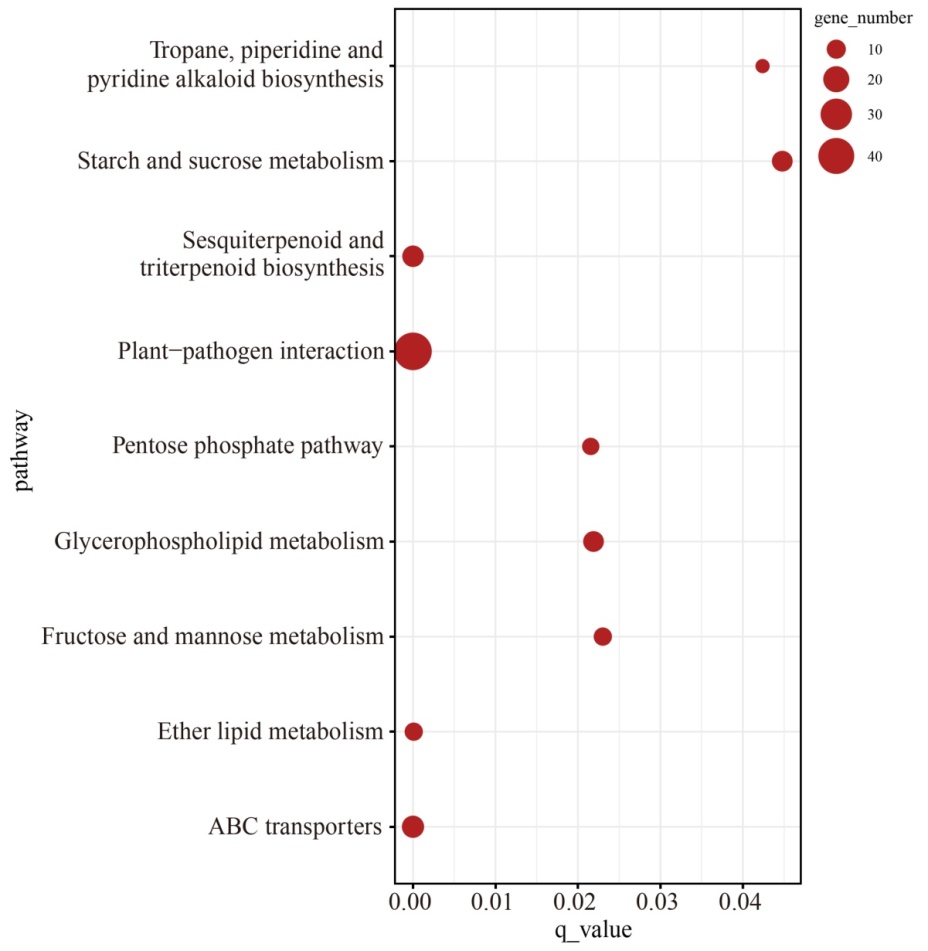


Figure S6 Kyoto Encyclopedia of Genes and Genomes (KEGG) enrichment analysis of contraction gene families in TJSM genome


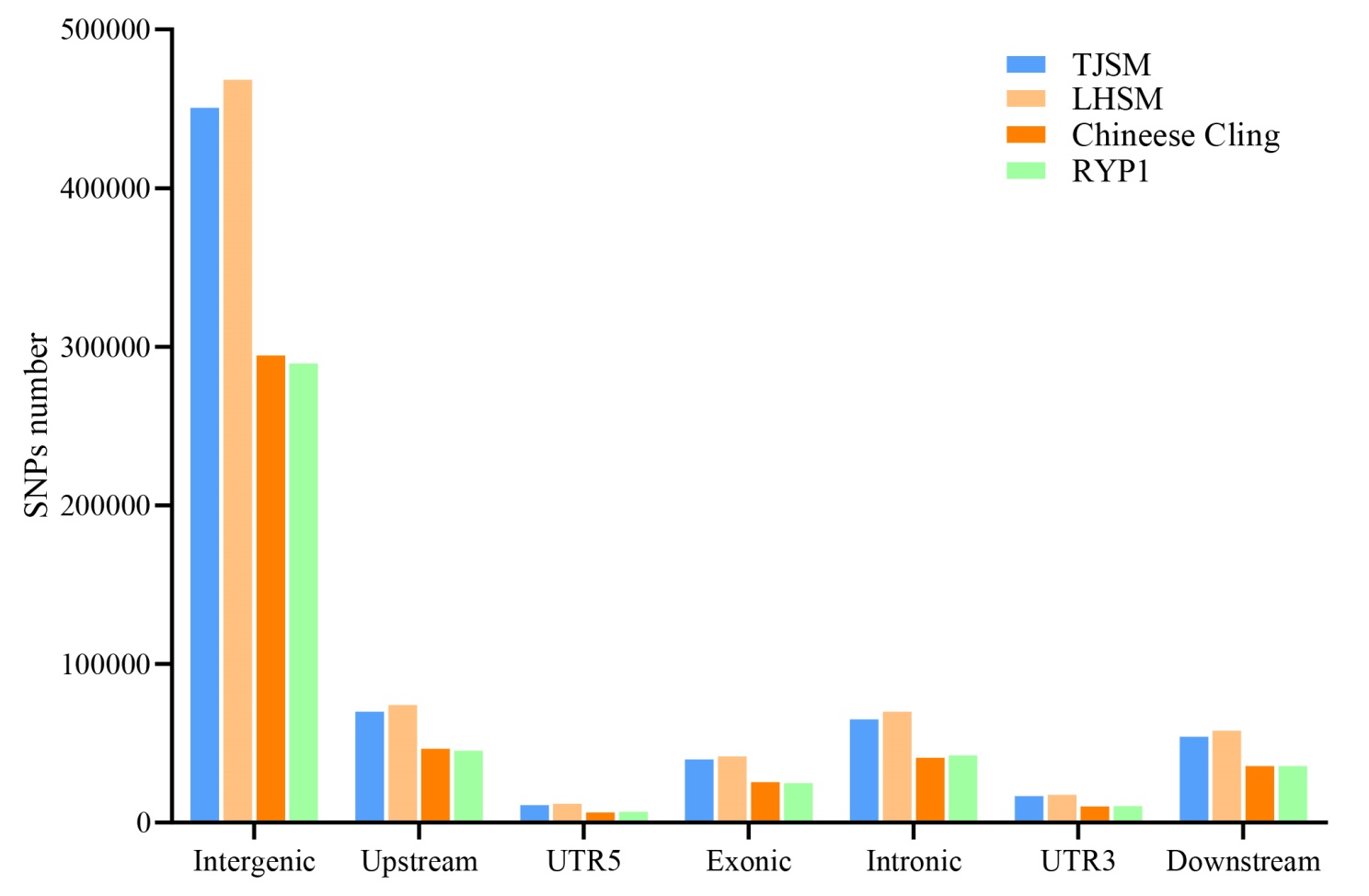


Figure S7 Gene distribution characteristics of SNPs in TJSM, Chinese Cling, LHSM, and RYP1 genomes


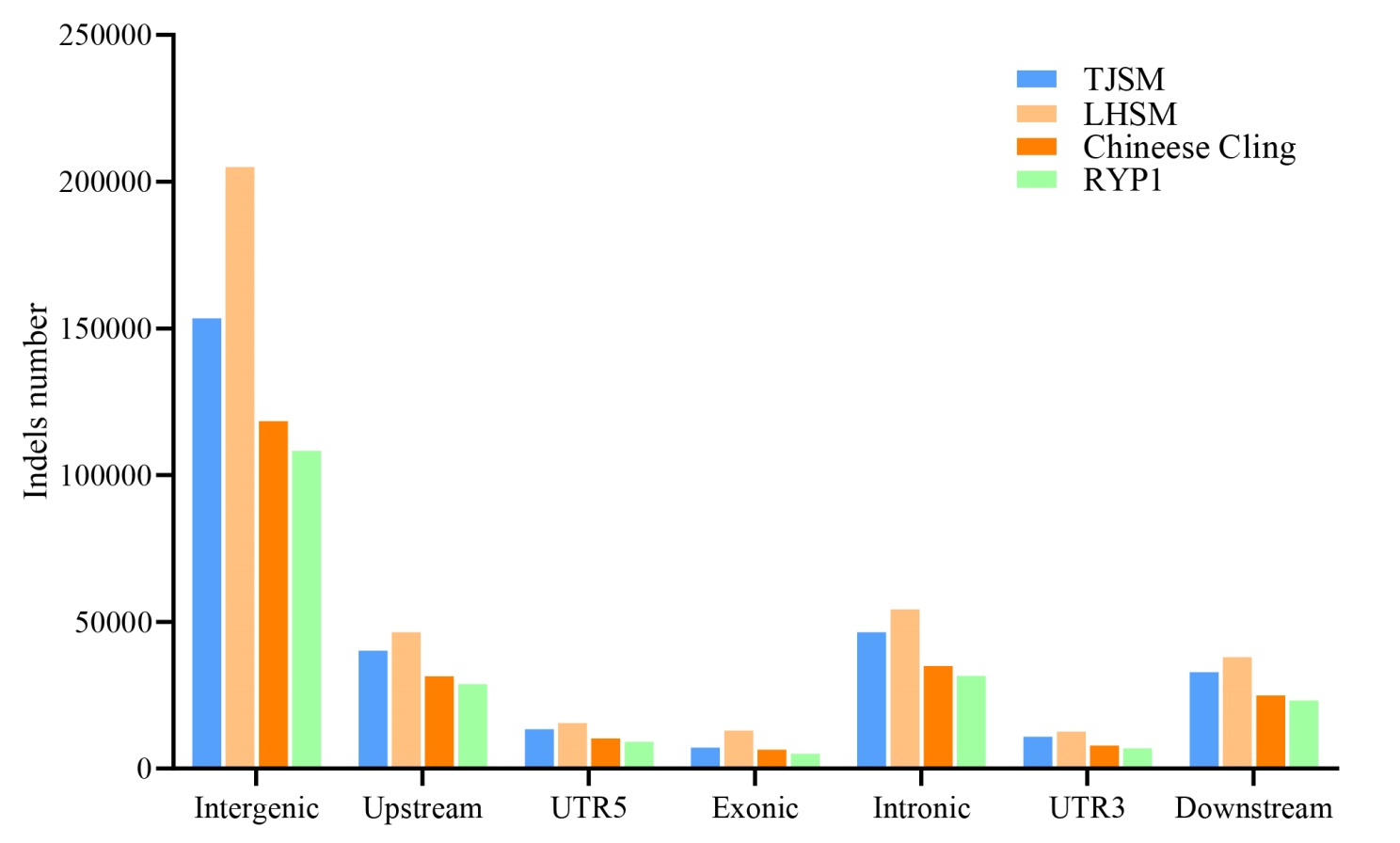


Figure S8 Gene distribution characteristics of Indels in TJSM, Chinese Cling, LHSM, and RYP1 genomes


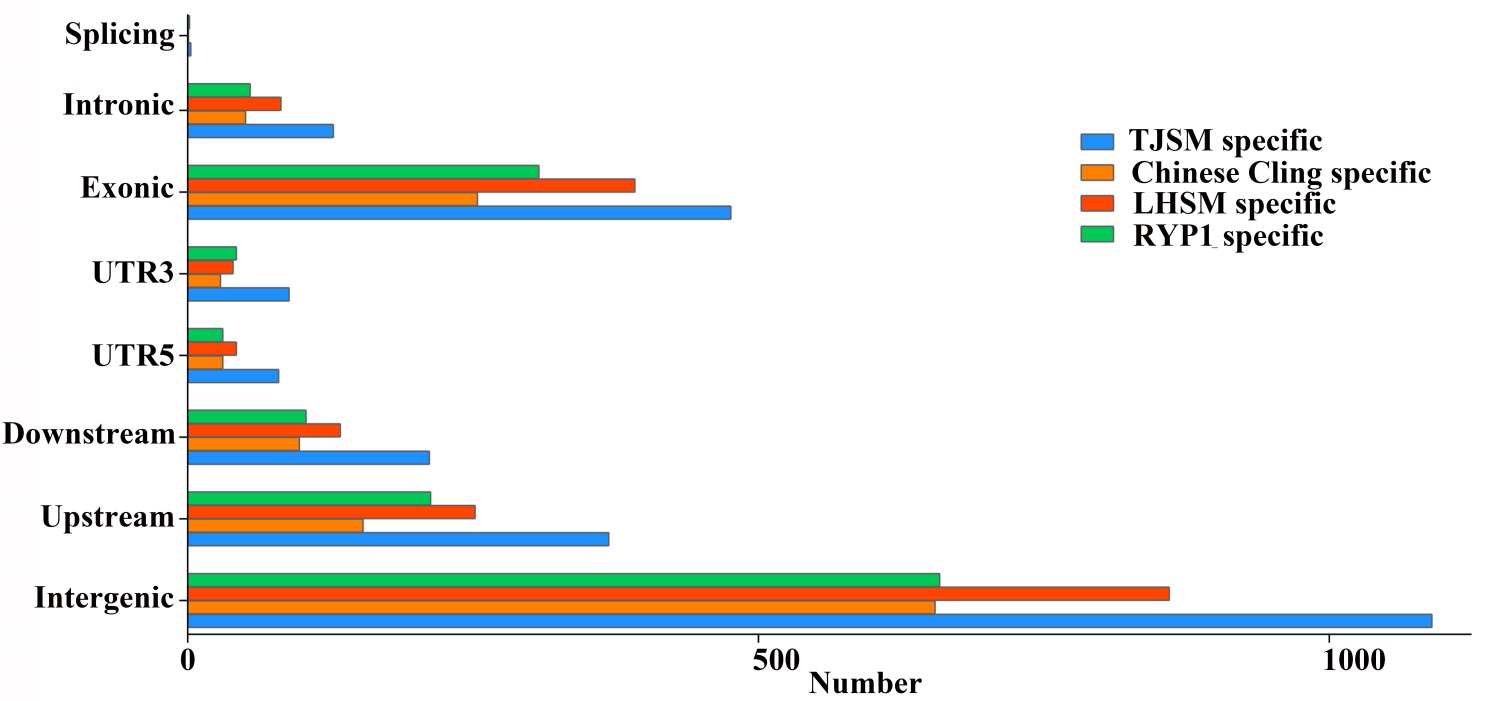


Figure S9 Gene distribution characteristics of specific SVs in TJSM, Chinese Cling, LHSM, and RYP1 genomes


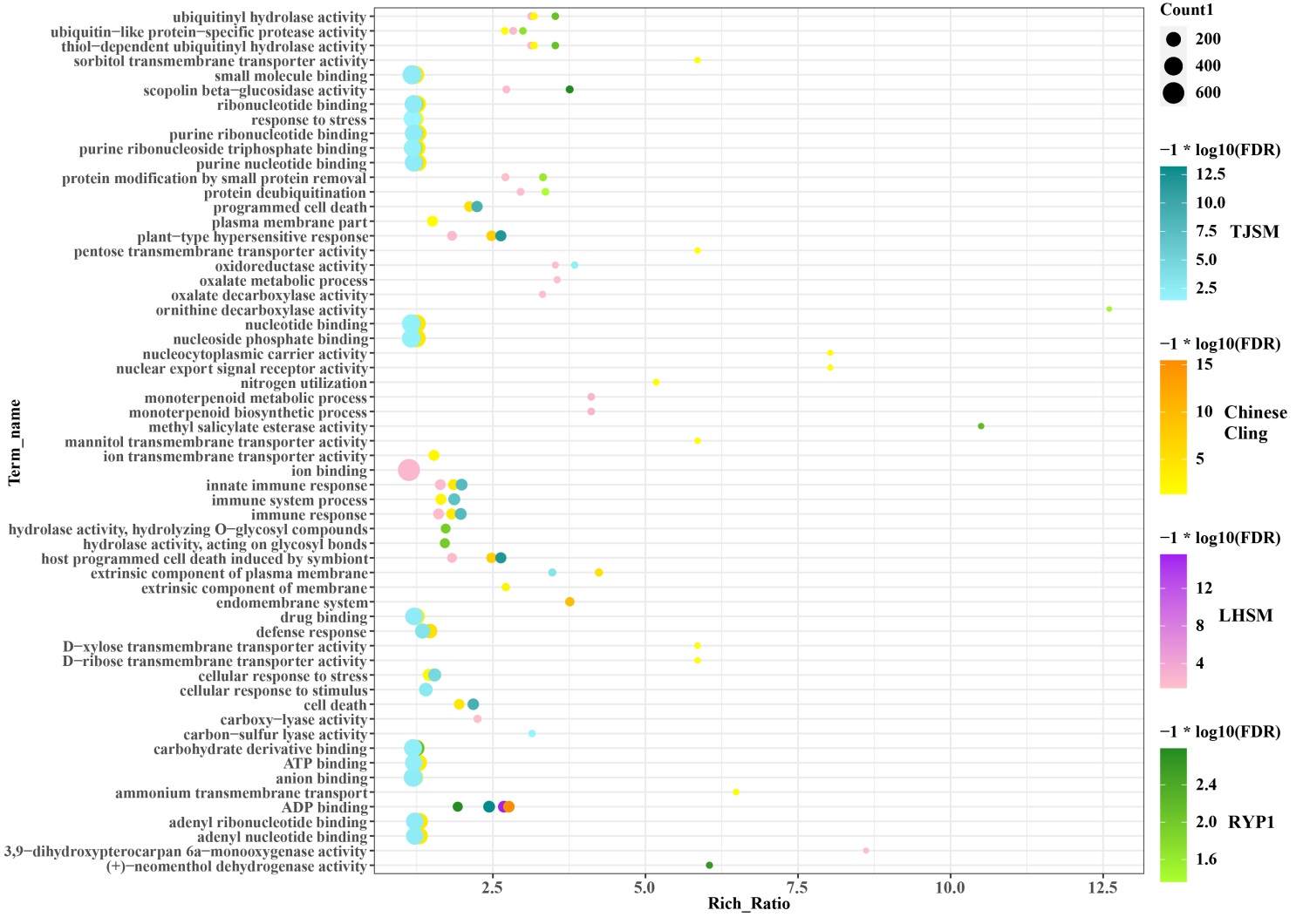


Figure S10 Gene ontology (GO) analysis of genes with specific SVs


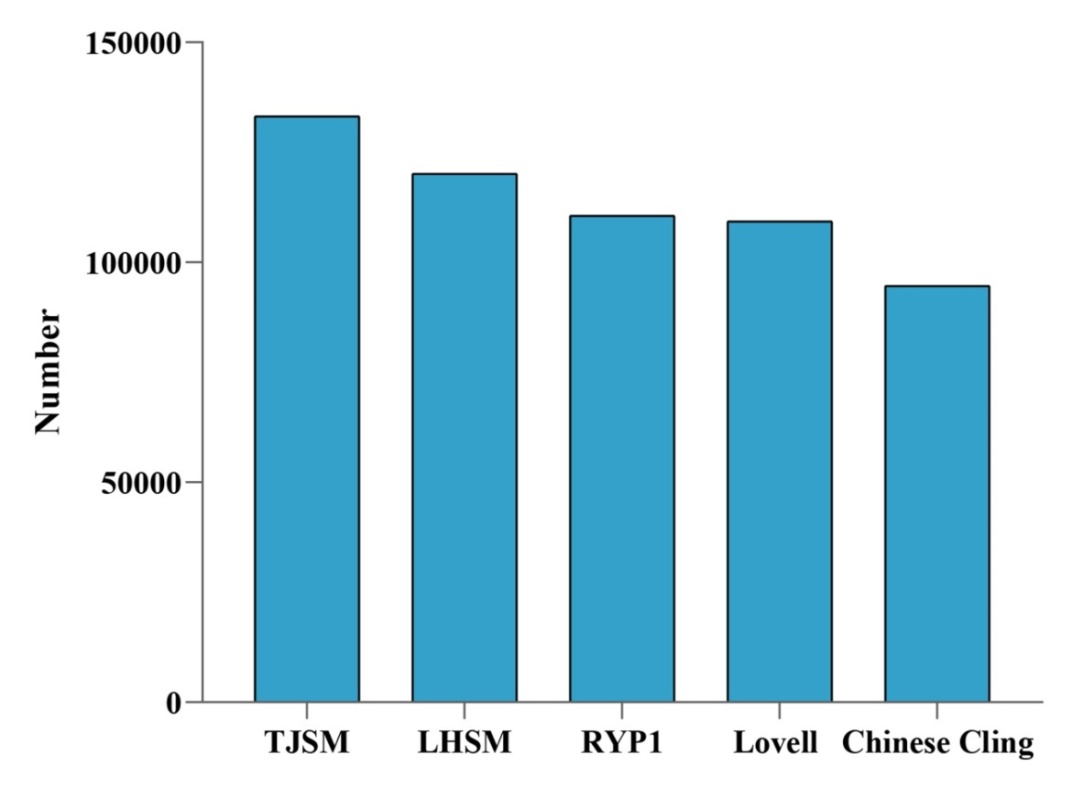


Figure S11 **N**umber of LTR-RTs in different genomes


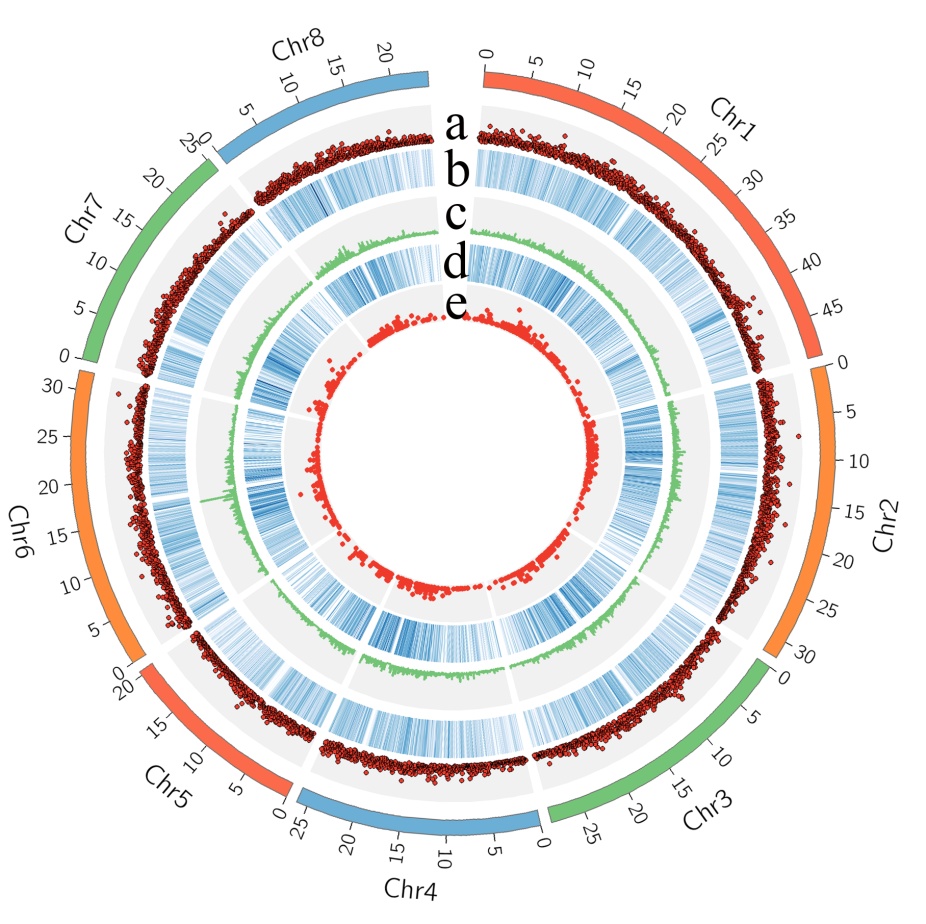


Figure S12 Distribution of different types of LTR-RTs in TJSM genome

Note: **a**, **b**, **c**, **d** and **e** represent type Ⅰ, type Ⅱ, type Ⅲ, type Ⅳ, and type Ⅴ of LTR-RT, respectively.

­
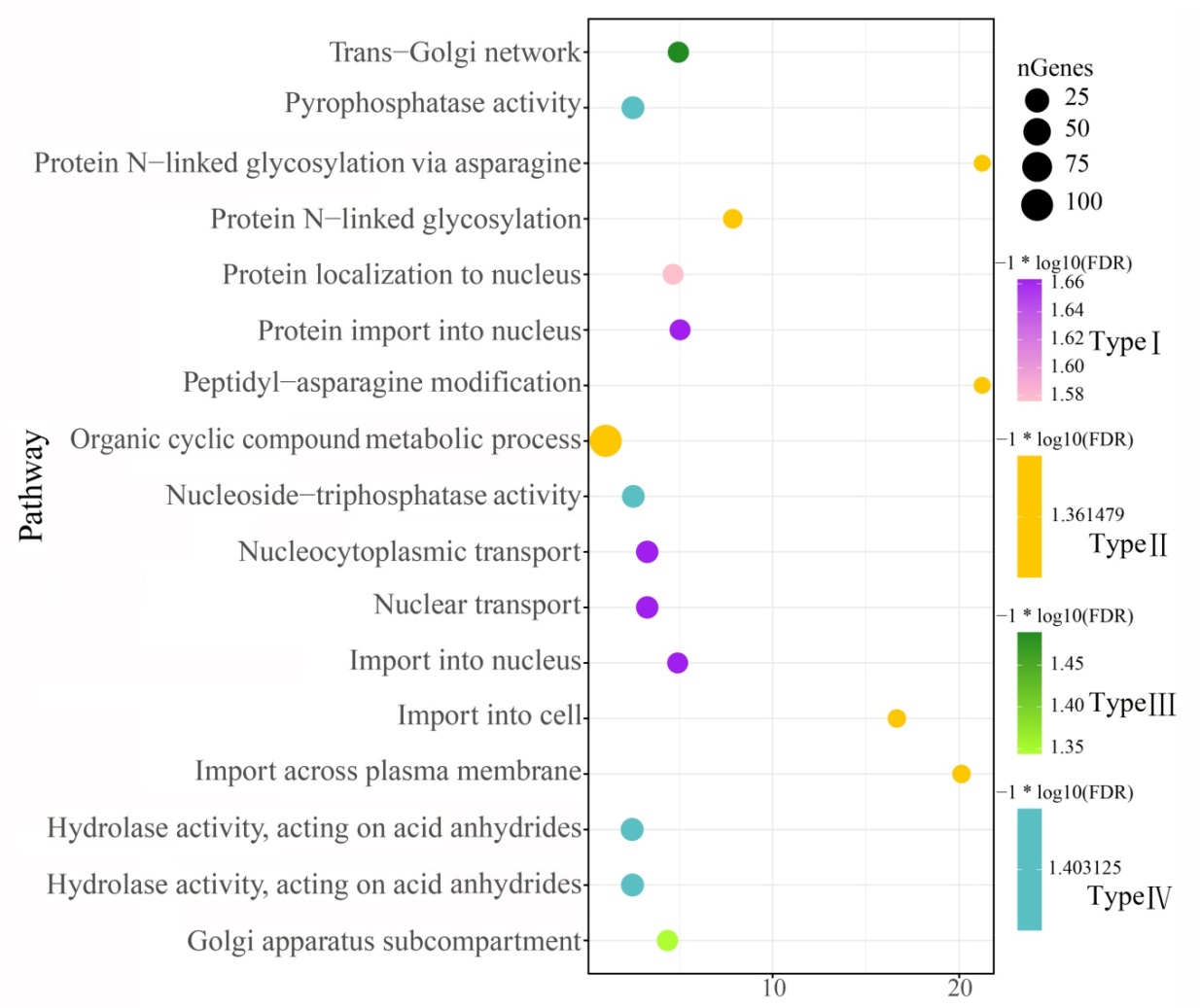


Figure S13 Gene ontology (GO) analysis of genes with different types of LTR-RT insertions


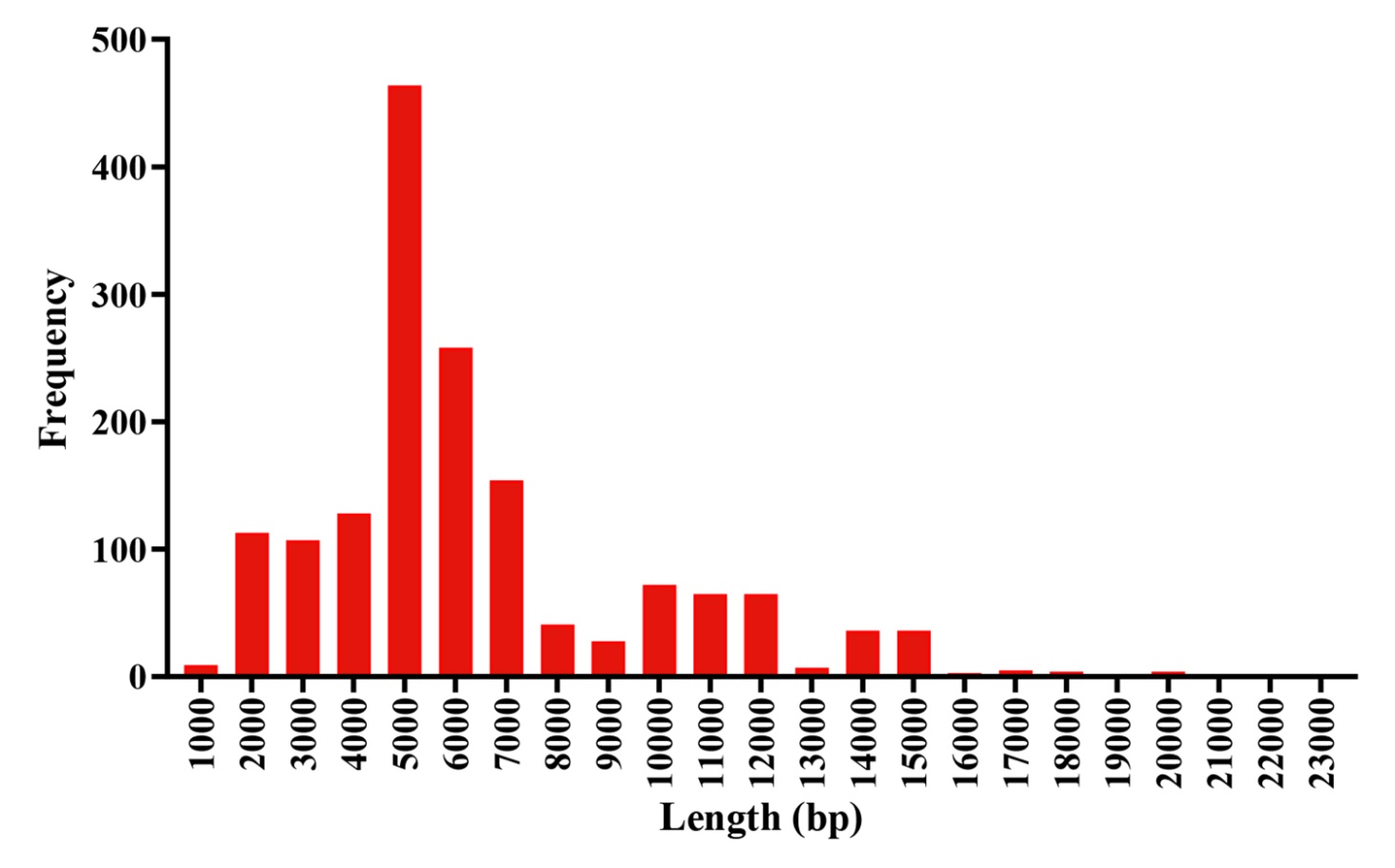
Figure S14 Different length of intact LTR-RTs identified from TJSM genome


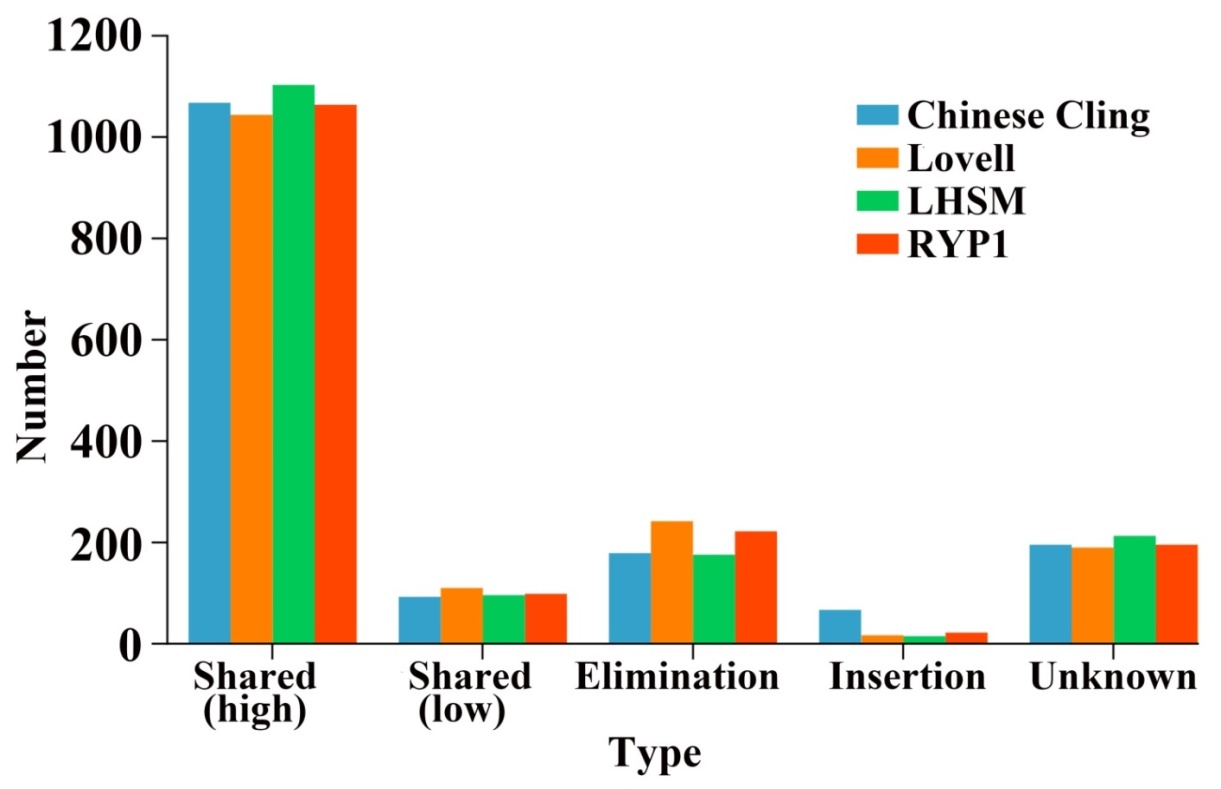


Figure S15 Classification of intact LTR-RTs by alignment of TJSM to Chinese Cling, Lovell, LHSM and RYP1 genome


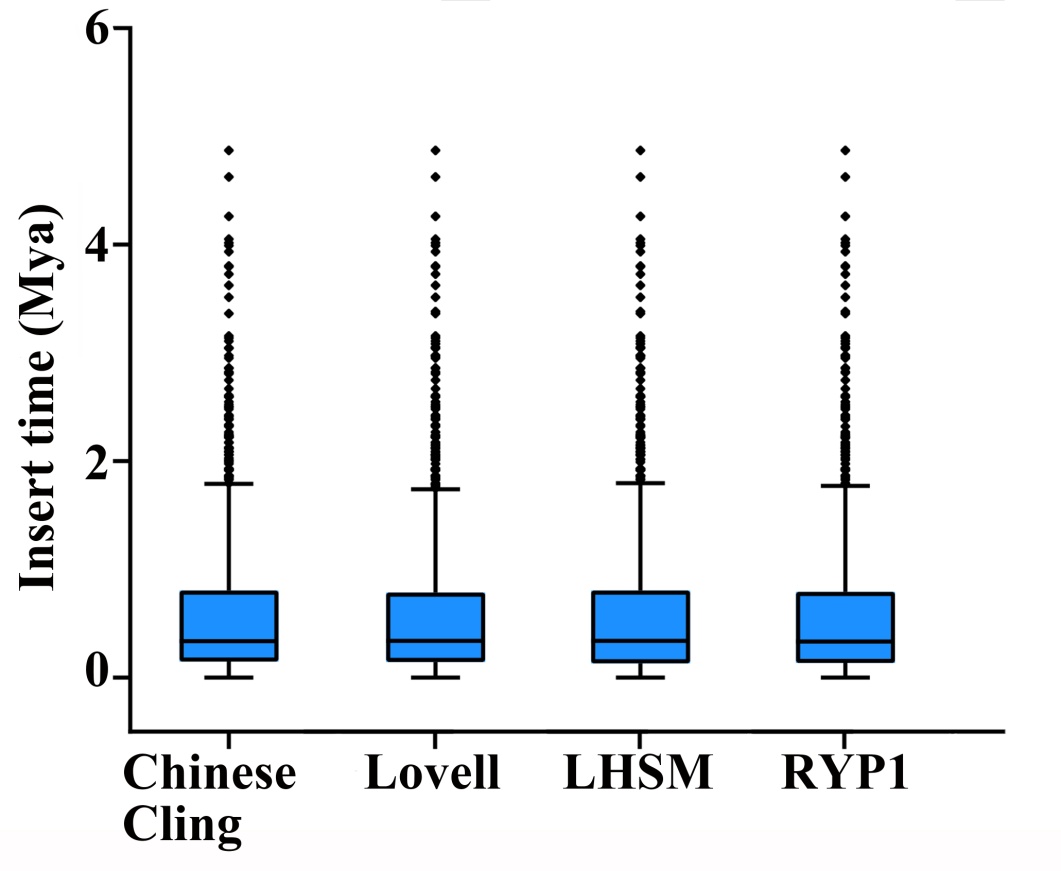


Figure S16 Estimated insert times of intact LTR-RTs shared by TJSM and four cultivated peach genomes reported


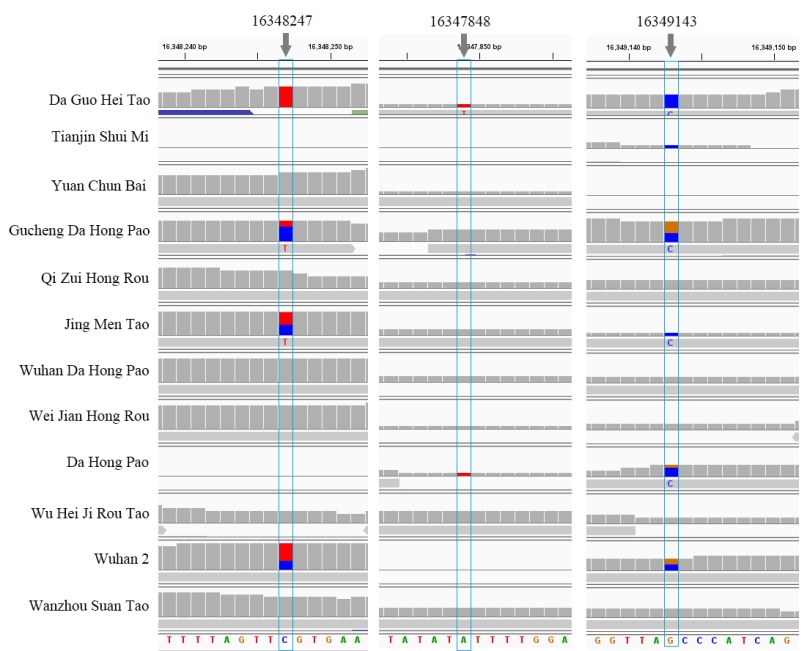


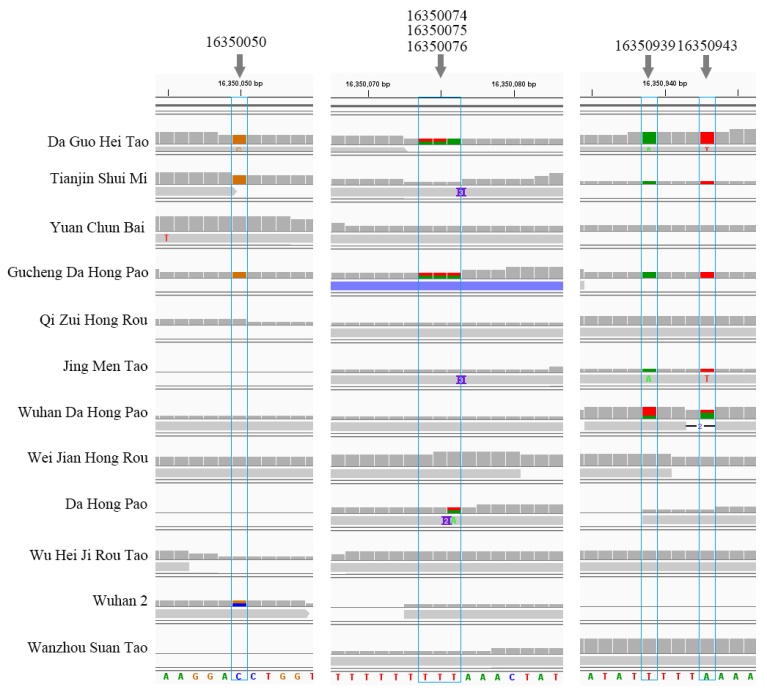


Figure S17 Detection of specific SNPs on *Prupe.5G203600* in blood flesh peach varieties


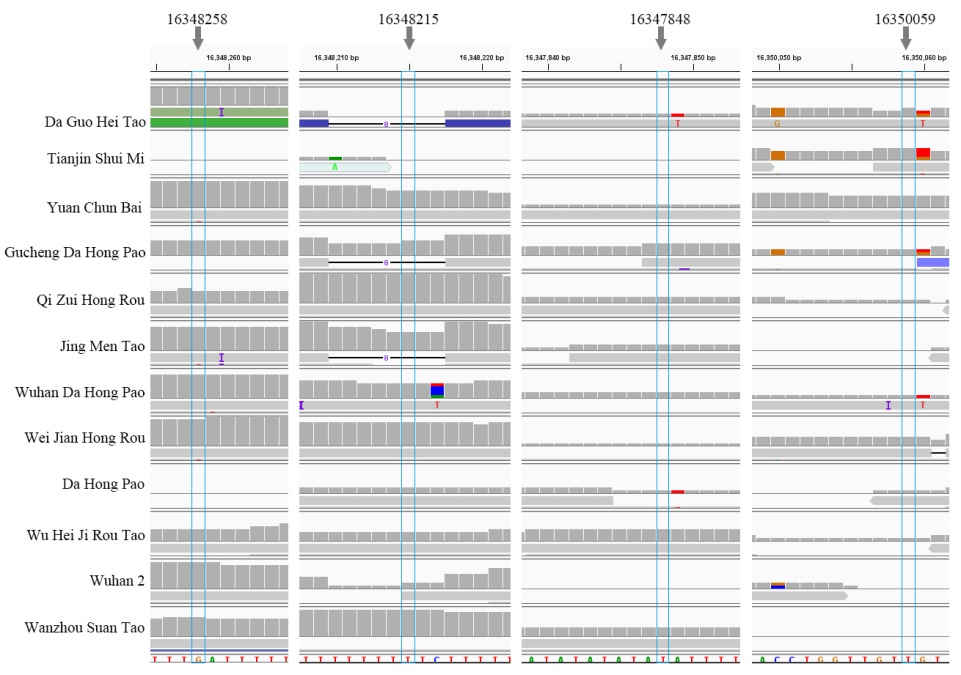


Figure S18 Detection of specific Indels on *Prupe.5G203600* in blood flesh peach varieties


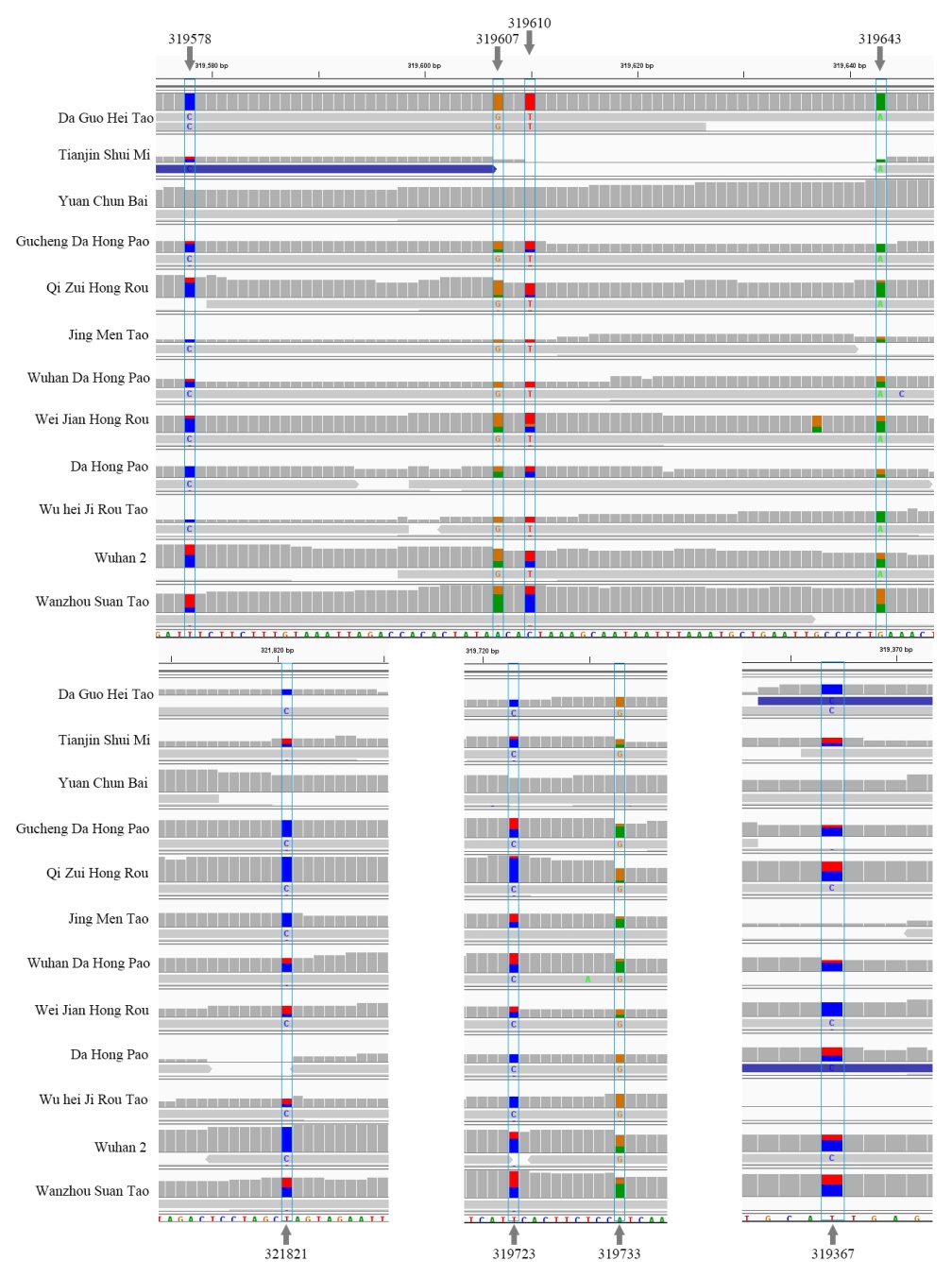


Figure S19 Detection of specific SNPs on *Prupe.1G003000* in blood flesh peach varieties


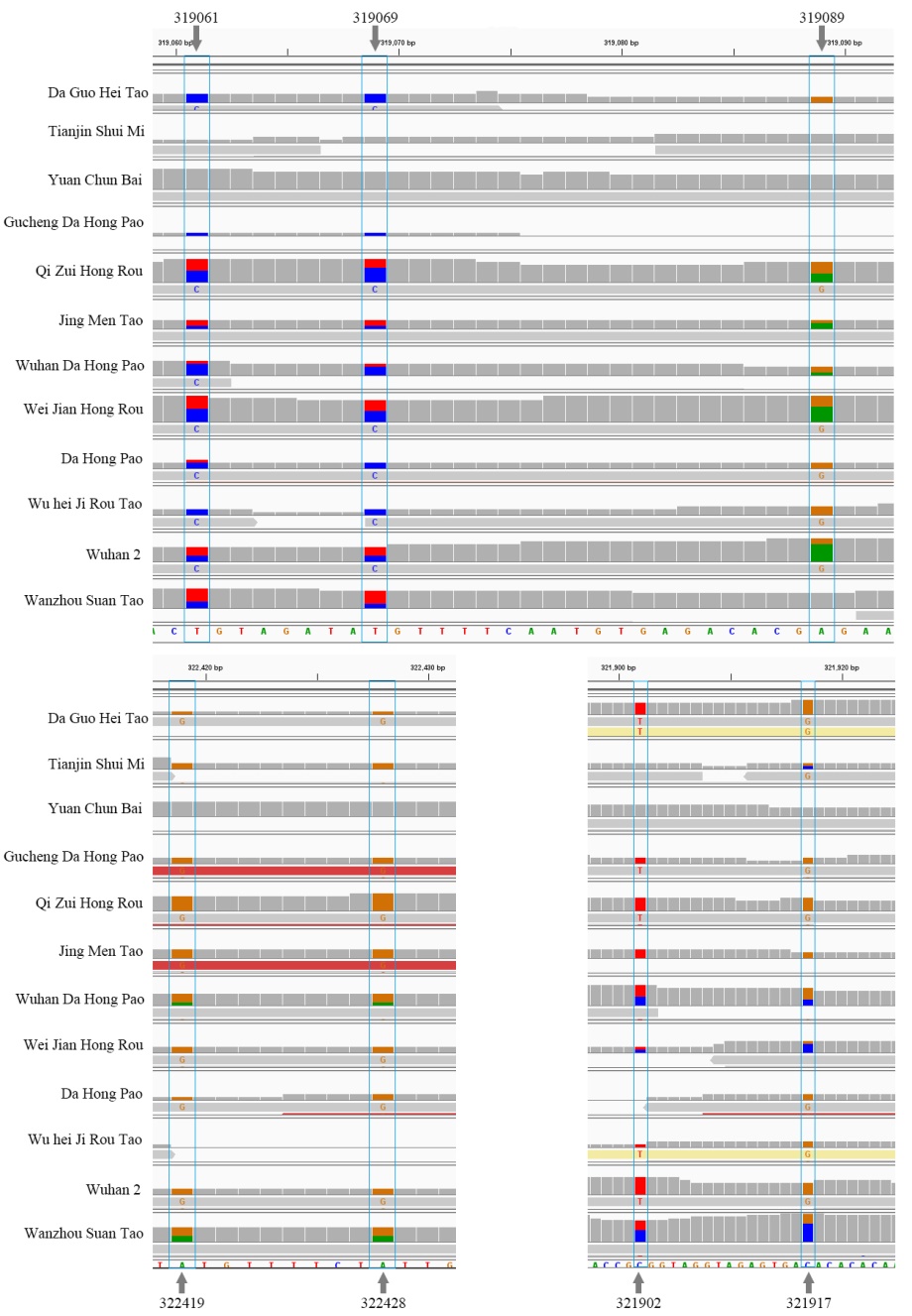


Figure S19 Detection of specific SNPs on *Prupe.1G003000* in blood flesh peach varieties


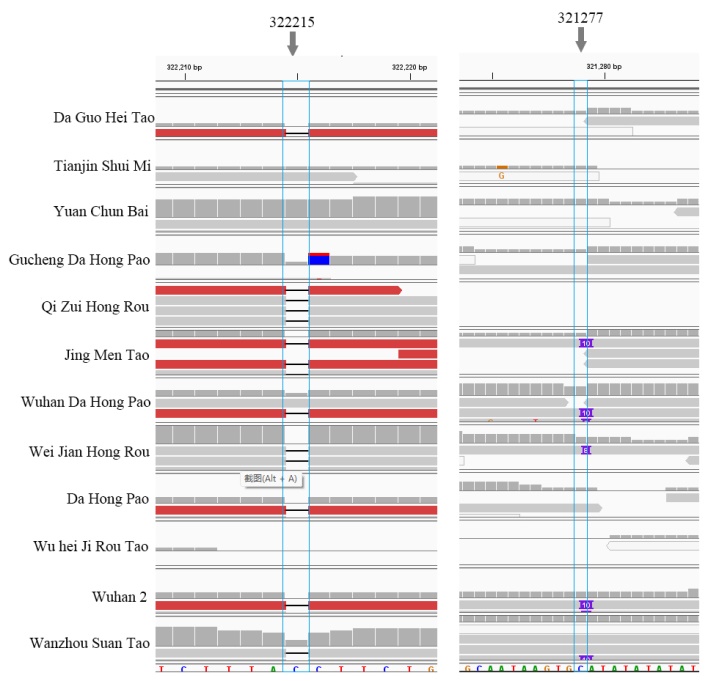


Figure S20 Detection of specific Indels on *Prupe.1G003000* in blood flesh peach varieties


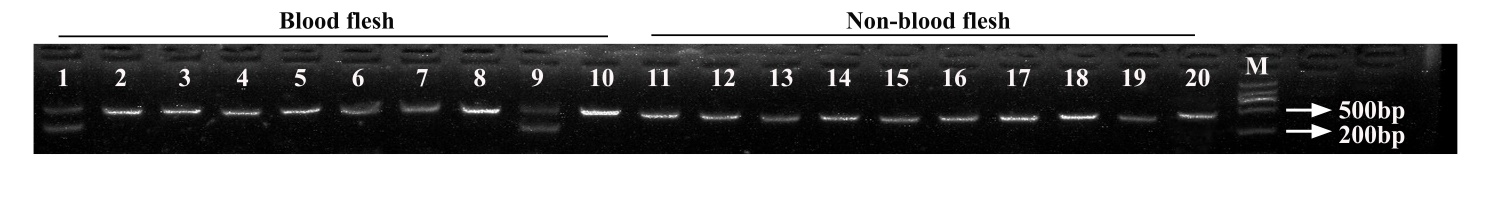


Figure S21 Correlation between genotype of 171bp deletion sequence on *Prupe.2G296600* and phenotype of fruit flesh color in 20 varieties

Note: The numbers from 1 to 10 and 11 to 20 indicate blood and non-blood flesh varieties (see Supplementary information Table 5). Long and short gel bands represent absence and presence of the deletion sequence on *Prupe.2G296600*, respectively.


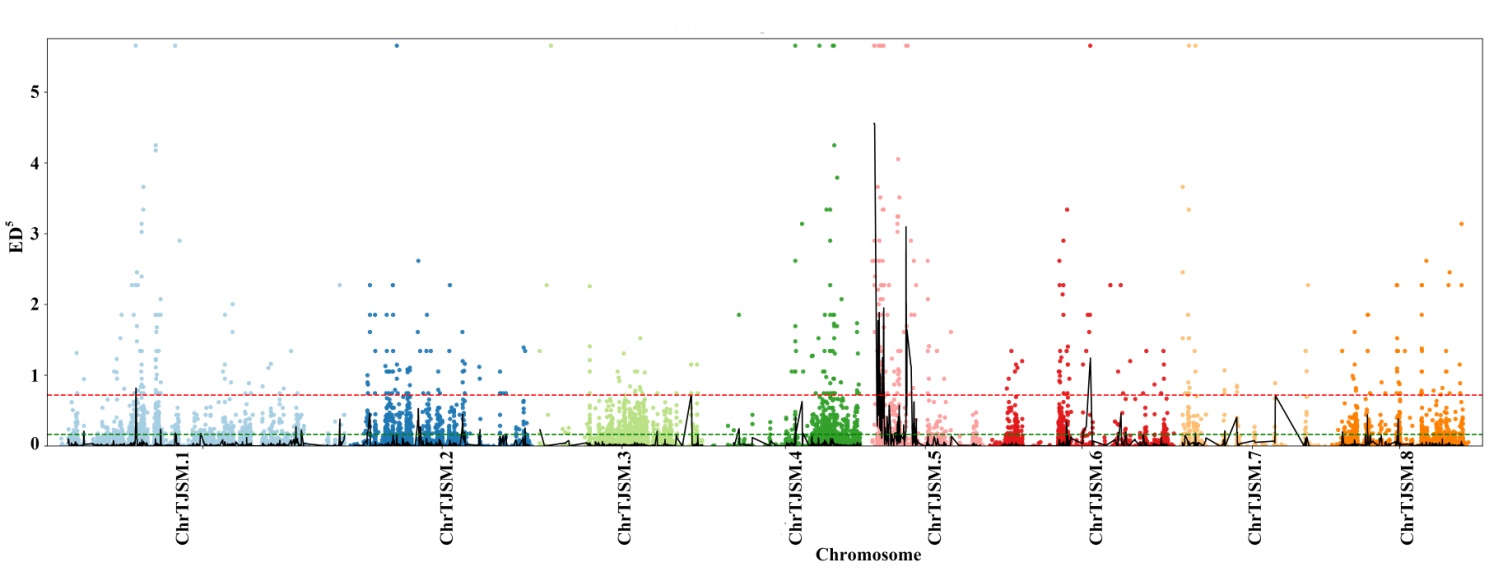


Figure S22 Localization of blood flesh traits based on BSA


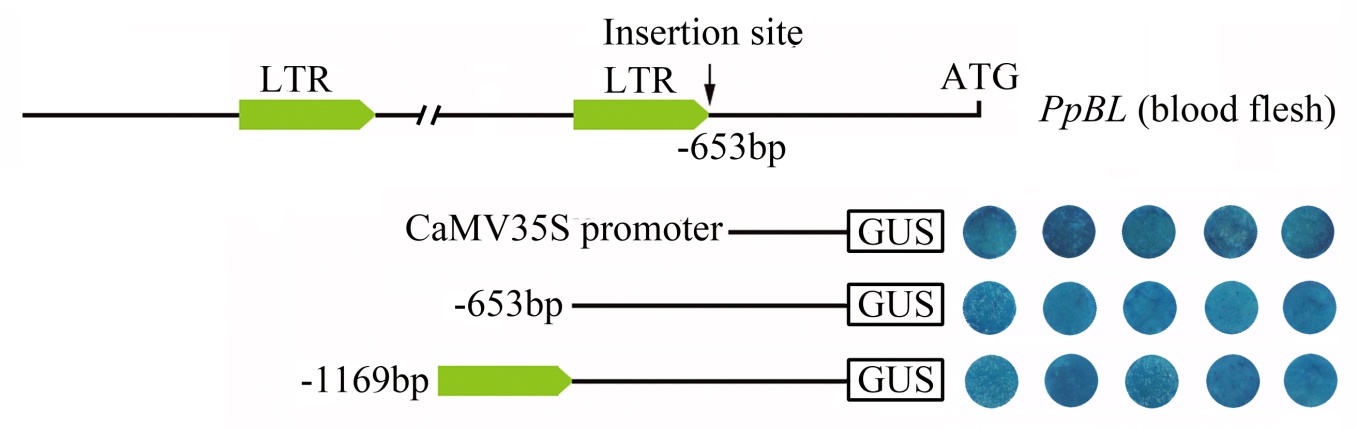


Figure S23 Activity of *PpBL* promoter with different lengths of sequences in transiently transfected peach fruit discs

Note: Promoter activity increases with increasing blue color intensity of peach fruit discs.


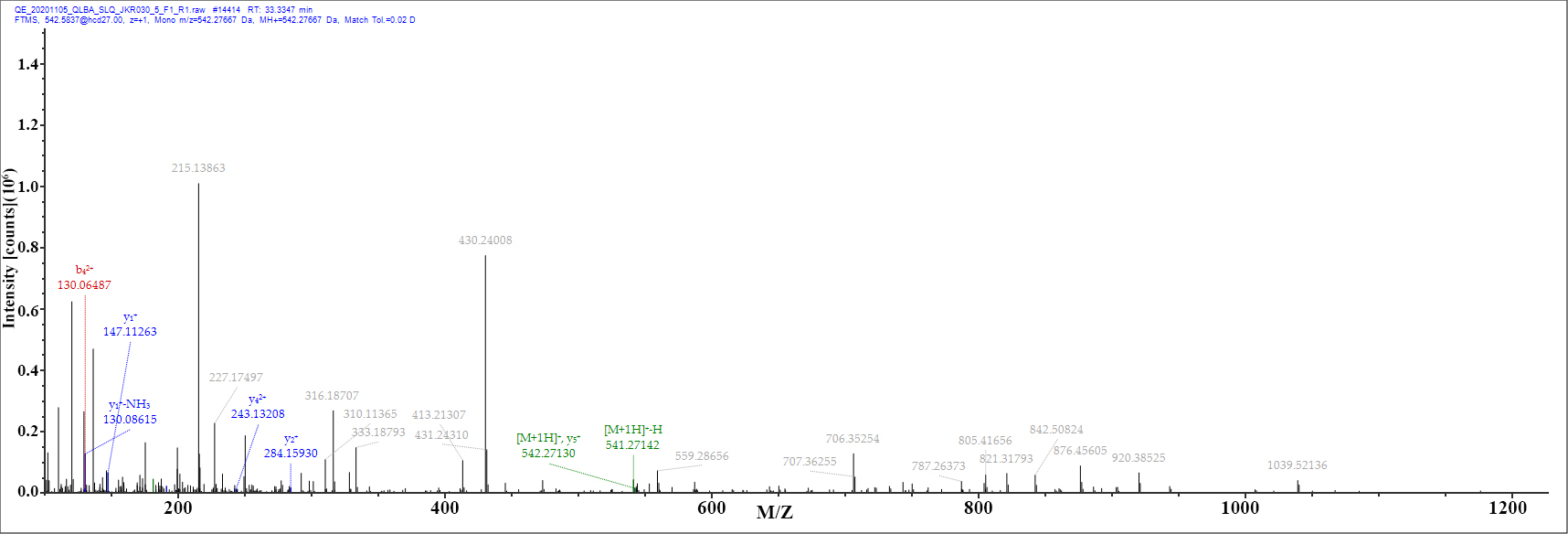


Figure S24 TIC total ions chromatogram corresponding to PpWRKY70


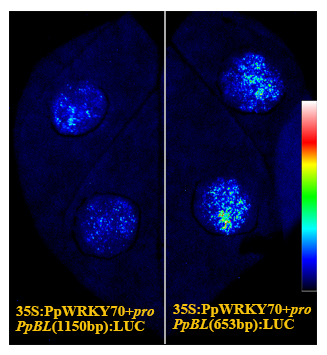


Figure S25 **Regulatory relationship of PpWRKY70 and *PpBL* promoter in transiently transfected *Nicotiana benthamiana* leaves**


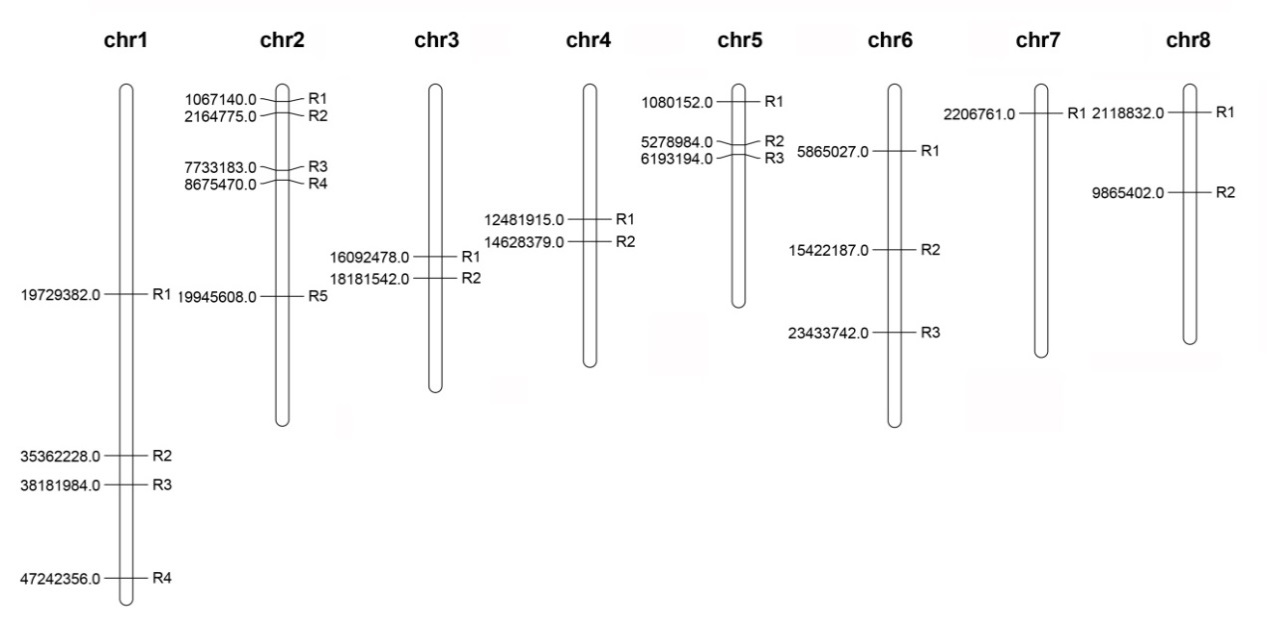


Figure S26 Distribution of homologous sequences of the blood TE on eight chromosomes
